# A mouse model with widespread expression of the C9orf72-linked glycine–arginine dipeptide displays non-lethal ALS/FTD-like phenotypes

**DOI:** 10.1038/s41598-022-09593-z

**Published:** 2022-04-04

**Authors:** Brandie Morris Verdone, Maria Elena Cicardi, Xinmei Wen, Sindhu Sriramoji, Katelyn Russell, Shashirekha S. Markandaiah, Brigid K. Jensen, Karthik Krishnamurthy, Aaron R. Haeusler, Piera Pasinelli, Davide Trotti

**Affiliations:** Jefferson Weinberg ALS Center, Department of Neuroscience, Vickie and Jack Farber Institute for Neuroscience, Thomas Jefferson University, Philadelphia, PA USA

**Keywords:** Neuroscience, Diseases of the nervous system, Neurological disorders, Motor neuron disease

## Abstract

Translation of the hexanucleotide G4C2 expansion associated with C9orf72 amyotrophic lateral sclerosis and frontotemporal dementia (ALS/FTD) produces five different dipeptide repeat protein (DPR) species that can confer toxicity. There is yet much to learn about the contribution of a single DPR to disease pathogenesis. We show here that a short repeat length is sufficient for the DPR poly-GR to confer neurotoxicity in vitro, a phenomenon previously unobserved. This toxicity is also reported in vivo in our novel knock-in mouse model characterized by widespread central nervous system (CNS) expression of the short-length poly-GR. We observe sex-specific chronic ALS/FTD-like phenotypes in these mice, including mild motor neuron loss, but no TDP-43 mis-localization, as well as motor and cognitive impairments. We suggest that this model can serve as the foundation for phenotypic exacerbation through second-hit forms of stress.

## Introduction

A hexanucleotide repeat G4C2 expansion in a noncoding region of the *C9orf72* gene is responsible for the most cases of amyotrophic lateral sclerosis (ALS) and frontotemporal dementia (FTD) of known genetic origin^1–3^. The expansion is hypothesized to elicit toxicity through both loss-of-function (LOF) and gain-of-function (GOF) mechanisms^4^. Haploinsufficiency, defined as reduction in expression of the endogenous C9orf72 (C9) protein, is the proposed LOF mechanism; the role of the C9 protein is still under investigation but growing evidence suggests that it acts as a Rab guanine exchange factor (GEF) when complexed with known autophagy-related proteins^5^. There are two proposed GOF mechanisms: (1) transcription of the repeat expansion leading to the formation of inherently toxic repeat-rich RNA species, and (2) translation of said RNA species to form toxic dipeptide repeat proteins (DPRs) in a process defined as repeat-associated non-AUG (RAN) translation^4^. Little is known about the interdependence of the three proposed pathogenic mechanisms; however, it has been shown that a reduction or loss of the C9 protein synergistically exacerbates DPR-mediated cellular toxicity^6^.

RAN translation results in the formation of five DPRs: poly-glycine–alanine (GA), glycine–arginine (GR), proline–alanine (PA), proline–arginine (PR), and glycine–proline (GP)^7,8^. Of these DPRs, the arginine-containing DPRs (poly-PR and poly-GR) are among the most toxic species^9^. The presence of DPRs has been implicated in multiple cellular events such as nucleocytoplasmic transport defects, phase-separation phenomena, and stress granule formation^10–12^. There is growing evidence demonstrating DPR-mediated toxicity in both in vitro and in vivo model systems where C9 protein haploinsufficiency and G4C2 repeat-rich RNA are intentionally absent, suggesting that the DPRs alone are sufficient to elicit a toxic profile in clinically relevant cell types^9,10,13–20^. Nevertheless, there is still much to learn about the singular contribution of DPRs to C9 disease pathogenesis.

Each DPR species has its own specific localization, aggregation-state, and toxicity profile^21^. Specifically, the DPR GR exists in both a diffuse and aggregated state and can compartmentalize in both the nucleus and cytoplasm. These different and oftentimes dynamic localization patterns confound the exploration of pathogenic mechanisms. Furthermore, it is accepted that at the level of the *C9orf72* G4C2 expansion, repeat lengths greater than 30 are associated with disease, suggesting a threshold for disease penetrance^22^. Evidence suggests that a similar length-dependency holds true at the level of the translated DPR. Specifically, in vivo model systems expressing GR with repeat lengths of 80 or greater display various patterns of survivability and phenotypic profile^13,14^. It remains unclear whether DPRs with lower repeat lengths confer a measurable toxicity in line with the penetrance patterns of the *C9orf72* expansion itself.

GR can be detected in human post-mortem tissue in clinically relevant central nervous system (CNS) areas (specifically cortex and spinal cord)^22^. This, combined with its dynamic localization profile and inherent toxicity in vitro, makes GR an ideal target to study the consequences of low-length DPR expression. In this study, we observe that a 50-repeat length GR (GR50) when expressed in primary neurons confers toxicity over an extended period. To study the expression of this DPR in vivo, we developed a novel mouse model in which GR50 was integrated using a knock-in FAST cassette system^23^ at the ROSA26 locus^24,25^, under the ROSA26 ubiquitous promoter system^25,26^. We identify central and peripheral nervous system-specific histopathological and functional differences in these mice. These phenotypes are associated with chronic mild neurotoxicity and a normal lifespan of the mice. We suggest that, alone, GR50 is sufficient to induce ALS/FTD-like motor and behavioral dysfunctions. Hence, we speculate that additional stressors and toxic mediators are likely required to intensify the motor and/or cognitive phenotypes associated with C9 ALS/FTD in this mouse model.

## Results

### Establishment of a novel mouse model expressing GR50

**Figure 1:**
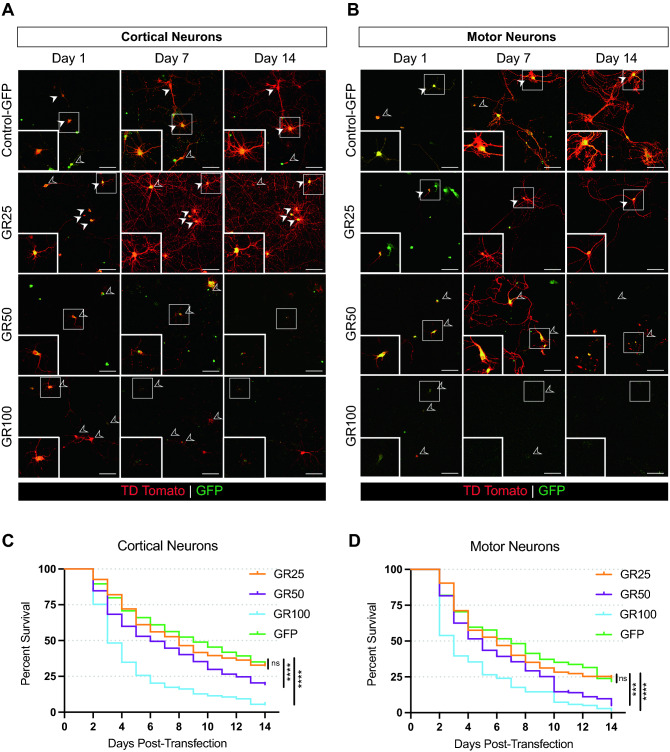
GR50 neurotoxic profile in vitro. Primary cortical neurons (**A**) and motor neurons (**B**) co-transfected with 125 ng td-tomato and 400 ng of GFP-tagged GR constructs of length 25, 50, 100, or GFP. Scale bar 100 µm. Inset shows cells that are double-positive for td-tomato and GFP-tagged construct of interest. Closed arrows indicate cells that survive over the 14-day observation period. Open arrows indicate cells that die over the time-course of the experiment. Log-rank longitudinal survival assessment of co-transfected td-tomato and GFP-GR25, 50, 100, or GFP alone quantified for primary corticals in (**C**) and motor neurons (**D**) over 14 days. Cells (m) pooled from n = 3 independent experiments. Primary cortical neurons m = 319 (GFP), 302 (GR25), 348 (GR50), and 263 (GR100). ****p < 0.0001. Primary motor neurons m = 149 (GFP), 105 (GR25), 163 (GR50), and 169 (GR100). ***p = 0.0001, ****p < 0.0001. Cell counts, p-values, and hazard ratios summarized in Tables 1 and 2.

Mouse models expressing GR > 80 display various disease phenotypic profiles^13,14^. However, it remains unclear whether lower repeat lengths confer a measurable toxicity, in line with the C9 expansion penetrance pattern^27–29^. To identify lower pathogenic GR lengths for in vivo evaluation, we first assessed the survival of GR-expressing cells in vitro. Previously, our lab has demonstrated length-dependent toxicity for different DPRs, including GR, expressed in cortical and motor neurons up to 7 and 5 days in vitro (DIV), respectively^9^. Within these time frames, we previously saw reduced survival in cortical neurons expressing GR of lengths greater than 100 repeats, as well as differing toxicity profiles between cortical and motor neurons. Interestingly, we later reported that heterologous expression of the DPR GA50 caused a delayed toxicity in cortical and motor neurons under observation for up to 14 DIV despite its lower repeat length^16^. This led us to question if this phenomenon held true for GR as well: if expressing GR with a lower number of repeats in cells observed over an extended time frame would also confer an overt toxic profile. To this end, we expanded our window of observation of GR-expressing cells to a total of 14 DIV. Cortical neurons and motor neurons were transiently transfected with a combination of eGFP or three different dipeptide lengths: GR25-, GR50-, and GR100-GFP, with a synapsin-driven cell-filling td-Tomato construct^9^. Cortical neurons (CNs) were transfected when deemed fully mature at DIV7, while motor neurons (MNs) were transfected at DIV5 as per our published paradigm^9^ (Supplementary Fig. 1). Cells were visualized daily for 14 days and those that were double-positive for GFP and td-Tomato fluorescence signal, indicating neuron-specific transfection, were assessed for survival. Indeed, with this expanded window of observation, a length dependent toxicity is observed in both cortical (Fig. 1C) and motor neurons (Fig. 1D), with GR100-eGFP-expressing cells showing the most rapid and robust reduction in survival. GR50-GFP-expressing cortical and motor neurons also display reduced survivability when followed over this extended time frame. This outcome is reflected in the loss of cortical and motor neurons over time, as shown in Fig. 1A,B. GR50-GFP-expressing cortical and motor neurons display a significantly higher risk of death (HR 1.455 for CNs, 1.529 for MNs) than cells expressing GR25-GFP (HR 0.9553 for CNs, 0.9883 for MNs) but not as robust as GR100-GFP-expressing cells (HR 2.441 for CNs, 2.062 for MNs) (Tables 1 and 2). We concluded that GR25 did not display a significantly different toxic profile compared to the eGFP control group, whereas GR100 showed rapid and robust neurotoxicity as also shown by HR values in both cortical and motor neurons. Based on this in vitro analysis we identified GR50 as a candidate dipeptide length for in vivo exploration with the rationale that its expression in vivo would indeed elicit chronic toxicity, but not so robustly that it would possibly prohibit a detailed longitudinal analysis and a temporal separation of different disease-related phenotypes.

**Table 1:** Statistical assessment of cortical neuron survival.

	# of cells	p-value	Hazard ratio	95% CI
GFP	319	Reference group		
GR25	302	0.6265	0.9553	0.7871–1.159
GR50	348	< 0.0001	1.455	1.218–1.739
GR100	263	< 0.0001	2.441	1.06–2.971

**Table 2:** Statistical assessment of motor neuron survival.

	# of cells	p-value	Hazard ratio	95% CI
GFP	149	Reference group		
GR25	105	0.9308	0.9883	0.7411–1.318
GR50	163	0.0001	1.529	1.202–1.946
GR100	169	< 0.0001	2.062	1.622–2.621

**Figure 2:**
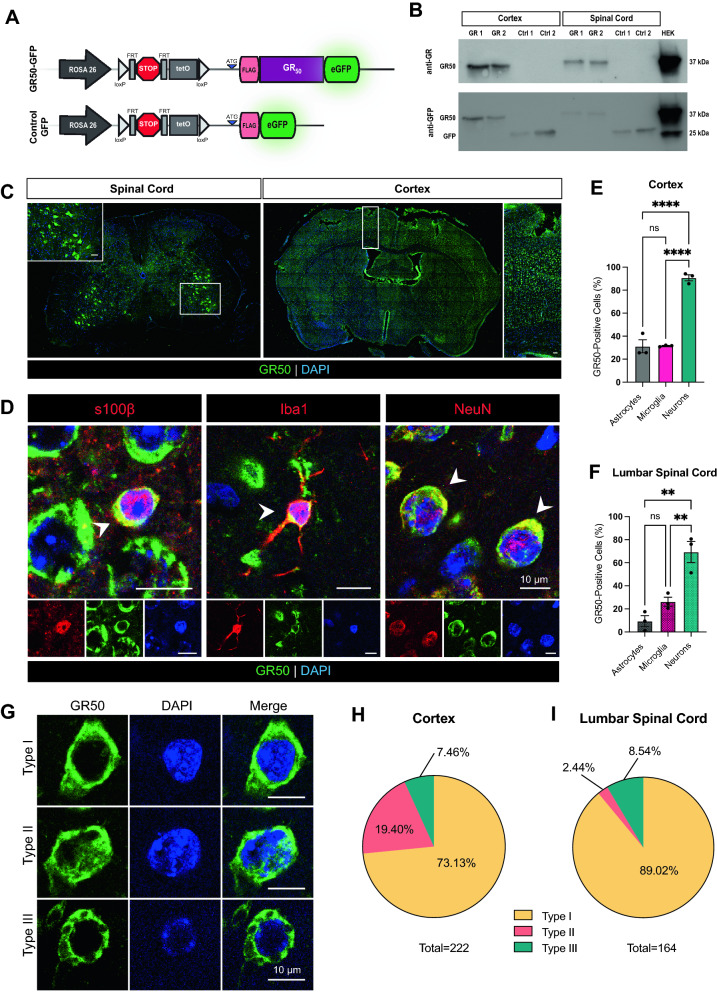
Establishment of in vivo mouse model of GR50 expression under a ubiquitous promoter system. (**A**) Schematic of the FAST cassette used to encode ATG-driven FLAG-GR50-GFP or FLAG-GFP in mice at the *Rosa26* locus under the ROSA26 promoter. (**B**) Representative western blot probing for GR (top) or GFP (bottom) in either GR50-GFP or control-GFP mice. Lysate from human embryonic kidney (HEK) cells transiently transfected with FLAG-GR50-GFP used as a positive control. (**C**) Representative image of endogenous expression of GR50-GFP in the lumbar spinal cord and cortex of a 3-month-old GR50-GFP mouse. Enlargement scale bars = 50 µm. Selected cortex area approximates region used for subsequent quantifications. (**D**) Representative images of S100β-, Iba1-, or NeuN-positive CNS cells expressing GR50-GFP in 3-month-old mice. Percentage of astrocytes, microglia, and neurons positive for GR50-GFP within cortical layers (**E**) and lumbar spinal cord (**F**). One-way ANOVA, Tukey’s multiple comparison’s test. (**E**) Cortex: n = 3 mice; mean ± s.e.m. Astrocytes vs. microglia p = 0.9972, neurons vs. astrocytes p < 0.0001, neurons vs. microglia p < 0.0001. (**F**) Lumbar Spinal Cord: n = 3 mice; mean ± s.e.m. Astrocytes vs. microglia p = 0.2246, neurons vs. astrocytes p = 0.0013, neurons vs. microglia p = 0.0071. (**G**) Representative images of the localization patterns of GR50 within CNS at 3-months-old. Type I: diffuse, cytosolic. Type II: cytosolic with evidence of perinuclear accumulation. Type III: cytosolic, aggregated. Percent distribution of localization types within the cortex and lumbar spinal cord quantified in (**H**), (**I**), n = 222 cells from cortex, 164 cells from lumbar spinal cord from 4 mice (2m, 2f). Localization types were scored manually with experimenter blinded to the genotype of the mice.

To determine whether GR50 expression could cause C9 ALS/FTD-related phenotypes in vivo, we employed a strategy that utilized a Flexible Accelerated Stop Tetracycline Operator (FAST) cassette system, which allows for multiple genetic manipulations under a single cassette^23^. The FAST cassette is inserted at the ROSA26 locus in C57BL/6 mice, a well-characterized safe-harbor site used for gene insertion^24,30^. This cassette relies on the ROSA26 promoter^25,26^; together, this combination facilitates ubiquitous expression of the FAST cassette transgene in mice. The ROSA26 promoter is a low-to-mild expression driver in comparison to other vertebrate promoters, including EF1a^31^. The FAST cassette includes a floxed STOP codon upstream of an ATG-driven FLAG-GR50-eGFP, or in the case of the control, FLAG-eGFP (Fig. 2A). When crossed with a constitutively active *Cre* mouse line that expresses Cre-recombinase driven by the ubiquitous promoter CAG (CAG-*Cre*), the STOP codon is excised, facilitating expression of the downstream FLAG-GR50-eGFP or FLAG-eGFP. In choosing this specific promoter system, we kept in mind that although our system expressed one DPR of one length, the C9orf72 expansion itself is ubiquitous in nature^32^. We also chose to use the ATG start codon ahead of FLAG in both the GR50-GFP and control-GFP mice so as not to rely on RAN-translation for protein expression. Furthermore, the GR50-encoding sequence consists of randomized alternative codons rather than the GGGGCC repeat sequence, eliminating the potential pathogenic effects of the formation of GC repeat-rich RNA transcripts. This design allowed us to assess the consequences of GR50 expression without intentionally introducing confounding LOF or other GOF disease mechanisms.

When crossed with CAG-*Cre* mice, the offspring that were heterozygous for both CAG-Cre and either FLAG-GR50-GFP (GR50-GFP) or FLAG-eGFP (control-GFP) were assessed. To ensure that our transgenic mice expressed the expected proteins of interest, we performed western blots using antibodies specific for GR or GFP on cortex and spinal cord tissue homogenates from 3-month-old mice. When a GR-specific antibody is used, we detect GR bands at approximately 37 kDa in the GR50-GFP mice, but not control-GFP animals (Fig. 2B, upper panel). This banding pattern is consistent with the one seen in HEK cell lysate transiently transfected with FLAG-GR50-GFP that we used as reference. When the same blot was stripped and re-probed, a GFP specific band is detected in both GR50-GFP and control-GFP mice at different heights as expected (Fig. 2B, lower panel). Control-GFP mice express a band at approximately 25 kDa while the band seen in GR50-GFP mice remains at approximately 37 kDa. Because the ROSA26 promoter confers ubiquitous expression, we also probed for GR or GFP expression in other CNS and non-CNS tissues of GR50-GFP and control-GFP mice (Supplementary Fig. 2). Homogenate from the cortex, cerebellum, spinal cord, and liver successfully show GR and GFP expression in GR50-GFP mice (Supplementary Fig. 2A) and positive GFP expression in control-GFP mice (Supplementary Fig. 2D). Interestingly, while GR50-GFP mice express relatively equal protein amounts across the neuronal tissues assessed, GR expression in the liver of GR50-GFP mice is significantly higher (Supplementary Fig. 2B,C). This higher liver expression is not readily observed in control-GFP mice, though equal expression across neuronal tissues is observed (Supplementary Fig. 2E). Nevertheless, liver tissue appeared healthy without visible signs of hepatotoxicity (Supplementary Fig. 2F).

In addition to understanding the expression pattern of GR50 across neuronal and non-neuronal tissues, we also wanted to understand the cell-type specific expression of GR50 across the CNS. We explored cell-type specificity by relying on the GFP fluorescence of GR50 in our mouse model, which we confirmed is expressed in CNS regions (Fig. 2C). We identified cells positive for both GR50-GFP and either s100β, iba1, or NeuN for astrocytes, microglia, and neurons, respectively (Fig. 2D). Throughout cortical layers, GR50-GFP is expressed in approximately 90.76% of neurons, 31.56% of microglia, and 31.20% of astrocytes (Fig. 2E). When visualizing transverse sections of lumbar spinal cord, GR50-GFP is expressed on average in 69.34% of neurons, 26.25% of microglia, and 9.42% of astrocytes (Fig. 2F).

Multiple groups have described the varied localization pattern of GR both in vitro^9^ and in vivo^13,14,22^, where GR exists in both a diffuse and aggregated form and can localize to both the nucleus and cytoplasm of an expressing cell. A detailed analysis of GR50-GFP expressing cells throughout the CNS in our GR50-GFP mice at 3 months of age identified three types of localization patterns, similar to classes described in a previous study^14^ (Fig. 2G–I). In our mouse, cells expressing GR50-GFP diffusely and localized to the cytoplasm (Type I cells) comprise of 73.13% of GR50-GFP expressing cells in the cortex and 89.02% in the lumbar spinal cord. Type II cells, while also demonstrating a diffuse, cytosolic localization, also show evidence of perinuclear accumulation as visualized in the nuclear folds, and comprise of 19.40% of GR50-GFP expressing cells in the cortex and 2.44% in the lumbar spinal cord. The remaining 7.46% of cells in the cortex and 8.54 in the cytosol are type III, consisting of aggregated GR50-GFP in the cytosol. While we did not reliably detect an aggregated, nuclear GR50-GFP signal, it is of note that endogenous poly-(GR) is expressed primarily in the cytoplasm of expressing CNS cells in patient tissue^22,33,34^, and the rare occasion of nuclear detection in patient tissue seems to be an artifact of non-specific antibody crossreactivity^22^. These localization patterns and frequencies differ from those observed in vitro (Supplementary Fig. 1C–E), as primary cortical and motor neurons transiently transfected with FLAG-GR50-GFP display the following localization parameters: Type A cells express a diffuse, cytosolic localization pattern much like type I cells in vivo, and comprise of 26.13% of primary cortical neurons and 25.13% of primary motor neurons. Most primary cortical and motor neurons are type B, consisting of a diffuse, cytosolic localization with evidence of nuclear aggregates, at 58.79% and 58.97%, respectively. Type C cells, much like type III cells in vivo, consist of cytosolic aggregates and is seen in 7.04% of primary cortical neurons and 8.21% of motor neurons. Type D cells consist of only nuclear aggregates and are observed in 8.04% of primary cortical neurons and 7.69% of primary motor neurons.

### GR50-expressing mice display sex-dependent histopathological differences

**Figure 3:**
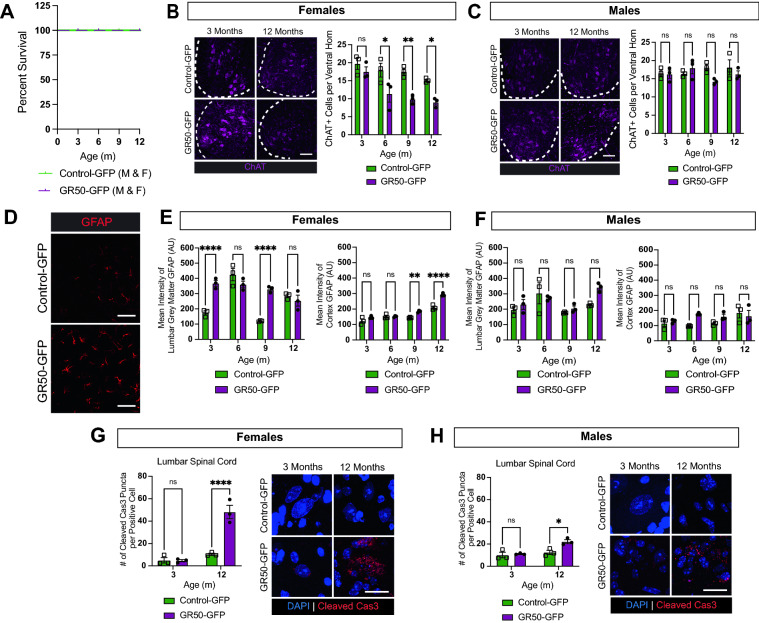
Histopathological changes in GR50 expressing mice. (**A**) Kaplan–Meier analysis of survival of GR50-GFP and control-GFP male and female mice over 12 months; n = 37 control-GFP mice, 29 GR50-GFP mice. (**B**) Representative image of ChAT-staining in the ventral horn of the lumbar spinal cord in female mice at 3 and 12 months. Scale bar = 100 µm. Quantification of chat-positive cells at 3-, 6-, 9- and 12-months. n = 3 mice per time-point, min. 6 fields per mouse for quantification, mean ± s.e.m. Two-way ANOVA, Sidak’s multiple comparisons test. 3-months p = 0.7329, 6-months p = 0.0163, 9-months p = 0.0051, 12-months p = 0.0269. (**C**) Same as in (**B**) but for male mice. 3-months p = 0.9997, 6-months p = 0.8340, 9-months p = 0.1198, 12-months p = 0.7119. (**D**) Representative image of GFAP fluorescence within the lumbar ventral grey matter in female control-GFP and GR50-GFP mice at 3-months-old. Scale bar = 50 µm (**E**) Quantification of lumbar ventral gray (left) and cortical GFAP expression (right) in female mice at 3-, 6-, 9- and 12-months-old. n = 3 mice per time-point, min 6 fields per mouse for quantification, mean ± s.e.m. Two-way ANOVA, Sidak’s multiple comparisons test. Female Lumbar: 3-months p < 0.0001, 6-months p = 0.2401, 9-months p < 0.0001, 12-months p = 0.8294. Female Cortex: 3-months p = 0.1654, 6-months p > 0.9999, 9-months p = 0.0059, 12-months p < 0.0001. (**F**) Same as in (**E**) but for male mice. Male Lumbar: 3-months p = 0.8662, 6-months p = 0.9017, 9-months p = 0.9442, 12-months p = 0.0551. Male Cortex: 3-months p = 0.9886, 6-months p = 0.0559, 9-months p = 0.4431, 12-months p = 0.9192. (**G**) Quantification of cleaved caspase-3 puncta accumulation within cleaved caspase-3 positive cells of the lumbar spinal cord in female control-GFP and GR50-GFP mice at 3- and 12-months-old. n = 3 mice per time-point, min 6 fields per mouse for quantification, mean ± s.e.m. Two-way ANOVA, Sidak’s multiple comparisons test. 3-months p = 0.9974, 12-months p < 0.0001. Right: representative images of cleaved caspase-3 puncta in control-GFP and GR50-GFP female lumbar spinal cord at 3- and 12-months-old. Scale bar = 20 µm. (**H**) Same as in (**G**) but for males. 3-months p = 0.9102, 12-months p = 0.0110. Right: representative images of cleaved caspase-3 puncta in control-GFP and GR50-GFP male lumbar spinal cord at 3- and 12-months-old. Scale bar = 20 µm.

We then investigated if GR50 expression could confer neurotoxicity in our mouse model. We assessed the consequences of said expression in CNS tissues from GR50-GFP mice compared to control-GFP mice. One of the most accepted pathological hallmarks in C9 ALS/FTD is the mis-localization of the nuclear protein TDP-43 to the cytoplasm in affected cells^35^. Importantly, the nuclear-to-cytoplasmic mis-localization of the ALS-relevant pathogenic marker TDP-43 is not robustly observed in GR50-GFP mice throughout the CNS at 12 months of age (Supplementary Fig. 4). We also did not observe any reduction in survival between sexes and genotypes in the test cohort over the course of 12 months (Fig. 3A). Neither female nor male GR50-GFP mice show evidence of robust neuron loss in the cortex as measured by NeuN expression compared to control-GFP mice between 3 and 12 months of age (Supplementary Fig. 3A,B). Female and male GR50-GFP mice also show no evidence of specific layer V motor neuron loss (Supplementary Fig. 3A,B).

While GR50-GFP females show no sign of overt neuron loss in the lumbar spinal cord at the same time-points as measured by NeuN expression (Supplementary Fig. 3D), there is a slight but significant reduction at 12 months in GR50-GFP males compared to control-GFP males (Supplementary Fig. 3E,F). These observations led us to assess cell-type specific differences in expression between GR50-GFP and control-GFP mice. As loss of motor neurons along the corticospinal tract is a hallmark of ALS pathogenesis, we investigated if motor neurons specifically were lost in GR50-GFP mice. When visualizing the ventral horn of lumbar spinal cord, we see a reduction in choline acetyltransferase (ChAT)-positive motor neurons in GR50-GFP female mice at 6, 9, and 12 months compared to control-GFP females (Fig. 3B). Interestingly, male GR50-GFP and control-GFP mice do not differ in the number of ChAT-positive motor neurons in the ventral horn at the same timepoints (Fig. 3C).

Sex-dependent differences are also seen at the level of astrocyte activation in CNS regions (Fig. 3D). Female GR50-GFP mice show increased glial fibrillary acidic protein (GFAP) activation in the ventral horn grey matter of the lumbar spinal cord at 3 months of age, prior to the time-points at which ChAT-positive cells were visibly lost (Fig. 3E). The cortex of female GR50-GFP mice expresses a higher GFAP signal compared to control-GFP females at later timepoints (Fig. 3E). Male GR50-GFP mice do not have increased GFAP activation in the lumbar ventral horn grey matter nor in the cortex compared to control-GFP males (Fig. 3F). Interestingly, between 3 and 12 months, we observe variable GFAP intensity in the spinal cord of both GR50-GFP and control-GFP females. Although this was not expected, dynamic GFAP signal along the course of disease has been observed in other neurodegenerative diseases^36^.

In addition to changes in GFAP expression and motor neuron counts, both males and females demonstrate increased evidence of the pro-apoptotic marker cleaved caspase-3 activity within the lumbar spinal cord as measured by cytosolic puncta accumulation over time^37–39^, correlating with some of the neuron loss observed in the spinal cord (Fig. 3G,H). However, cleaved caspase-3 accumulation is not seen in the cortex of male and female mice (Supplementary Fig. 3G,H).

### Differences in sciatic nerve function absent of demyelination are observed in female GR50-expressing mice

**Figure 4:**
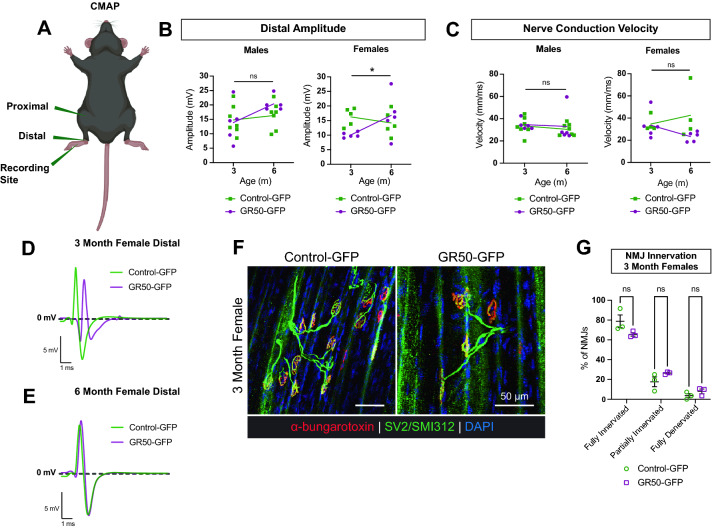
Female mice demonstrate CMAP functional differences absent of abnormal NMJ morphology. (**A**) Schematic of CMAP stimulating and recording electrode placement along the sciatic nerve within the hindlimb of test mice. (**B**) Distal amplitude recordings in male (left) and female (right) control-GFP and GR50-GFP mice at 3- and 6- months old. Males: n = 5 GR50-GFP, 7 control-GFP; time x genotype p = 0.2385. Females: n = 5 mice per genotype; time x genotype p = 0.0386. Two-way ANOVA, Sidak’s multiple comparisons test. (**C**) Nerve conduction velocity in male (left) and female (right) control-GFP and GR50-GFP mice at 3- and 6- months old. Males: n = 5 GR50-GFP, 7 control-GFP; time x genotype p = 0.8522. Females: n = 5 GR50-GFP, 4 control-GFP; time x genotype p = 0.7671. Two-way ANOVA, Sidak’s multiple comparisons test. (**D**) Representative distal CMAP stimulus trace of a female control-GFP and GR50-GFP mouse at 3 months and (**E**) 6 months. Dashed line indicates 0 mV. (**F**) Representative images of the neuromuscular junctions of the extensor digitorum longus muscle of female control-GFP and GR50-GFP mice at 3-months-old stained for presynaptic SV2 and postsynaptic alpha-bungarotoxin. Scale bar = 50 µm. (**G**) Quantification of the innervation status of NMJs of the soleus and extensor digitorum longus muscles of female control-GFP and GR50-GFP mice at 3-months-old. n = 3 mice per time-point, mean ± s.e.m. Two-way ANOVA, Sidak’s multiple comparisons test. Fully Innervated: p = 0.0717, Partially Innervated: p = 0.3016, Fully Denervated: p = 0.7910.

We next assessed if the histopathological consequences of GR50 expression along with the observed sex-specificity translated into functional consequences reminiscent of ALS progression. As motor unit dysfunction and loss correlates with ALS progression, we recorded the compound muscle action potential (CMAP) along the sciatic nerve in GR50-GFP and control-GFP mice as a measure of motor unit integrity and function^40^ (Fig. 4A). Waveform amplitude was measured as an indication of the number of functional nerve fibers and degree of muscle bulk, while the nerve conduction velocity was used as evidence of myelination state, as reduction in nerve conduction velocity may indicate a loss of signal-insulating myelin^41^.

We performed CMAP recordings in a cohort of male and female GR50-GFP and control-GFP mice at two different time-points corresponding with the initial onset of motor neuron loss in female GR50-GFP mice (3 versus 6 months old). In males, both genotypes show no difference in the amplitude when stimulated from the distal site (Fig. 4B) at both the 3-month and 6-month time-point, comparable with the lack of specific motor neuron loss observed in male mice. Distal amplitude is reduced in 3-month-old GR50-GFP females compared to control-GFP females, suggestive of distal axonal dysfunction at this age (Fig. 4B,D). At 3 months, however, female mice do not demonstrate significant differences in neuromuscular junction (NMJ) integrity, as shown by the degree of presynaptic synaptic vesicle 2 (SV2) and postsynaptic α-bungarotoxin signal overlap (Fig. 4F,G). In both male and female GR50-GFP and control-GFP mice, no difference in nerve conduction velocity is observed (Fig. 4C), suggesting that demyelination did not contribute to observed deficits.

Interestingly, when the same cohort of female GR50-GFP mice underwent 6-month CMAP recordings, the deficit in distal amplitude is no longer observed and is comparable to control-GFP females (Fig. 4B,E). This observation could suggest a developmental delay as opposed to a pure distal axonal deficit, or motor neuron compensation since we report a reduction in ChAT-positive neurons in 6-month-old female mice. When a separate cohort of GR50-GFP and control-GFP females was evaluated at 12-months-old, there was still no difference in either the proximal or distal amplitudes nor the nerve conduction velocity, nor was there a difference in NMJ innervation state (Supplementary Fig. 5).

### Mice expressing GR50 display sex-dependent differences in gait dynamics

When examining GR50-GFP and control-GFP animals, it was difficult to discern the genotype as the mice appear indistinguishable. This observation, combined with the functional and histopathological differences observed led us to hypothesize that there may be subtle, but measurable, locomotor differences that could be quantified. Impaired gait is one locomotor measurement that may not be so obvious to the experimenter unless the gait discrepancies were overt. Changes in gait dynamics have been observed in ALS patients in conjunction with their decline in motor function^42,43^, therefore we considered this an appropriate metric to measure in our mouse model to identify a more subtle relevant phenotype.

**Figure 5:**
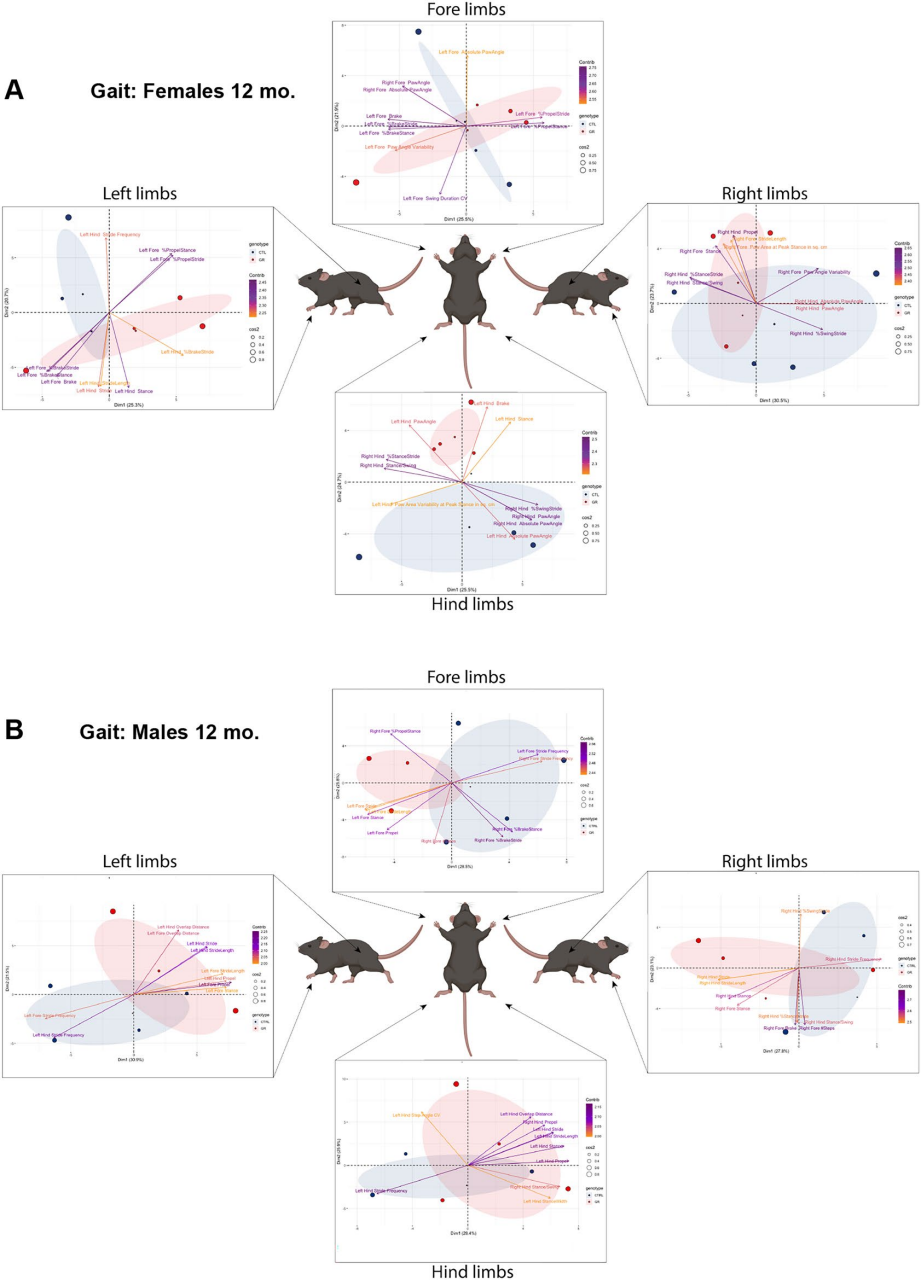
PCA identifies difference in hindlimb gait dynamics. Principal component analysis of the Digigait gait performance of left, right, fore, and hindlimbs in female (**A**) and male (**B**) control-GFP (blue) and GR50-GFP (red) mice at 12 months old. n = 4 mice per sex and genotype. Ellipses indicate spread of variance. Size of dot representing each mouse defines its overall contribution to the spread of the variance. Arrows list top 10 Digigait measurements, or indices, that contribute to the principal component space. The color of each arrow is scaled according to the indices’ contribution to the first two dimensions of the principal component space (Contrib. purple-orange scale).

Gait analysis was performed on GR50-GFP and control-GFP animals using the Digigait quantitative gait assessment apparatus. The mice briefly ran on a clear treadmill to gather video footage of their steps. This footage was then analyzed for over 40 measurable gait indices, or features, associated with each of the four limbs. Because we aimed to understand if an overall difference in the gait of GR50-GFP and control-GFP mice could be measured rather than a change in a particular feature, we performed a principal component analysis (PCA) as a dimension reduction tool to identify trends and groupings among the genotypes. A PCA performed on Digigait data generated from the male and female mice at 12-months-old again shows a sex and genotype-specific difference in gait dynamics. The underlying variance of GR50-GFP females naturally separates from control-GFP only in the hindlimb gait features, indicative of a distinct difference in the hindlimb gait dynamics of GR50-GFP females compared to controls (Fig. 5A). Males, however, do not demonstrate a natural separation in variance for any limbs, suggesting that gait differences could not be detected between male GR50-GFP and control-GFP animals (Fig. 5B).

### Mice expressing GR50 display a subtle motor and behavioral deficit

**Figure 6:**
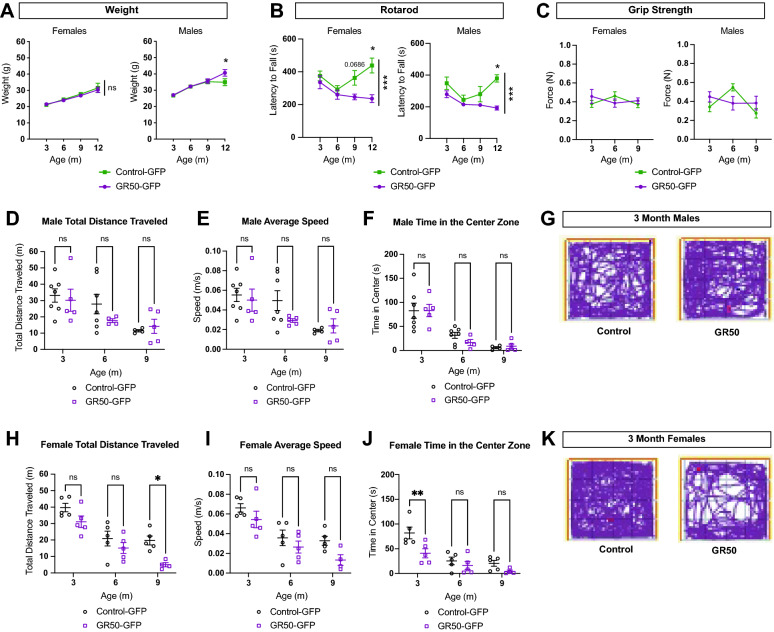
Motor and behavioral phenotypes in GR50 expressing mice. (**A**) Weight of female (left) and male (right) control-GFP and GR50-GFP mice at 3, 6, 9, and 12 months. Females: control-GFP n = 15, 15, 13, 6; GR50-GFP n = 14, 11, 11, 6. Males: control-GFP n = 12, 13, 11, 5; GR50-GFP n = 14, 11, 11, 6. Mean ± s.e.m. Females: 3-months p = 0.9945, 6-months p = 0.9934, 9-months p = 0.9184, 12-months p = 0.9373. Males: 3-months p = 0.9997, 6-months p > 0.9999, 9-months p = 0.9976, 12-months p = 0.0473. (**B**) Rotarod performance of female (left) and male (right) control-GFP and GR50-GFP mice at 3, 6, 9, and 12 months. Females: control-GFP n = 14, 9, 9, 4; GR50-GFP n = 14, 11, 11, 6. Males: control-GFP n = 12, 13, 10, 5; GR50-GFP n = 10, 9, 8, 3. Mean ± s.e.m. Females: 3-months p = 0.8103, 6-months p = 0.9430, 9-months p = 0.0686, 12-months p = 0.0177. Males: 3-months p = 0.1998, 6-months p = 0.8804, 9-months p = 0.3002, 12-months p = 0.0117. (**C**) Hindlimb grip strength measurements of female (left) and male (right) control-GFP and GR50-GFP mice at 3, 6, and 9 months. Females: n = 5 per genotype; Males: n = 7 control-GFP, 5 GR50-GFP. Mean ± s.e.m. Females: time x genotype p = 0.4314. Males: time x genotype p = 0.5490. (**D**) Male total distance traveled in Open Field Assessment. Control-GFP n = 7, 7, 4; GR50-GFP n = 5, 4, 5. Mean ± s.e.m. 3-months p = 0.9654, 6-months p = 0.4424, 9-months p = 0.9810. (**E**) Male average speed in Open Field Assessment. Control-GFP n = 7, 7, 4; GR50-GFP n = 5, 5, 5. Mean ± s.e.m. 3-months p = 0.9535, 6-months p = 0.2208, 9-months p = 0.9759. (**F**) Male time spent in the center zone in Open Field Assessment. Control-GFP n = 7, 7, 4; GR50-GFP n = 5, 4, 5. Mean ± s.e.m. 3-months p > 0.9999, 6-months p = 0.7245, 9-months p = 0.9974. (**G**) Representative track plots of male control-GFP and GR50-GFP mice at 3-months. (**H**) Female total distance traveled in Open Field Assessment. Control-GFP n = 5, 5, 5; GR50-GFP n = 5, 5, 4. Mean ± s.e.m. 3-months p = 0.1890, 6-months p = 0.5160, 9-months p = 0.0158. (**I**) Female average speed in Open Field Assessment. Control-GFP n = 5, 5, 5; GR50-GFP n = 5, 5, 4. Mean ± s.e.m. 3-months p = 0.4798, 6-months p = 0.6563, 9-months p = 0.1392. (**J**) Female time spent in the center zone in Open Field Assessment. Control-GFP n = 5, 5, 5; GR50-GFP n = 5, 5, 4. Mean ± s.e.m. 3-months p = 0.0056, 6-months p = 0.8140, 9-months p = 0.4907. (**K**) Representative track plots of female control-GFP and GR50-GFP mice at 3-months. For all datasets, Two-way ANOVA with Sidak’s multiple comparisons test was used for statistical analysis.

Finally, we sought to determine if GR50 expression translates to a measurable motor and behavioral phenotype in mice. Prior to behavioral assessments, animals were weighed to determine if potential weight loss should be considered a confounding factor. While female mice do not show a difference in weight over time between genotypes, GR50-GFP males display increased weight gain compared to control-GFP males at 12-months-old (Fig. 6A). Mice were subject to motor assessment using an accelerated rotarod performance task. Both male and female GR50-GFP mice have a reduced latency to fall compared to control-GFP animals (Fig. 6B). While rotarod performance is affected in GR50-GFP expressing animals, neither male nor female hindlimb grip strength show significant differences at 3, 6, and 9 months of age (Fig. 6C). Nevertheless, while sex-dependent histopathological and functional differences are observed, both sexes expressing GR50-GFP display a locomotive deficit.

As C9orf72-ALS lies on a genetic and clinical spectrum with FTD, we also tested whether expression of GR50 correlated with a measurable cognitive impairment. We relied on the open field assessment to identify both changes in locomotion and evidence of general anxiety behavior. Male GR50-GFP mice do not significantly differ from control-GFP males in the total distance traveled in the chamber, their average speed in the chamber, or the time they spent in the center of the chamber at 3, 6, and 9-months-old (Fig. 6D–G). Female GR50-GFP and control-GFP mice do not significantly differ in their average speed, though GR50-GFP females trend lower at those same timepoints (Fig. 6I). GR50-GFP expressing females travel a shorter total distance in the chamber at 9-months-old than control-GFP females (Fig. 6H). At 3-months-old, GR50-GFP female mice spend less time in the center zone of the chamber, evidence of increased anxiety compared to control-GFP females of the same age (Fig. 6J,K).

## Discussion

In this study, we identify GR50 as a potential pathogenic DPR despite its shorter repeat length both in vitro and in vivo*. *In vitro*,* we establish GR50-mediated toxicity over a 14-day observation period in primary cortical and motor neurons. Prior to this study, we understood that GR50 expression did not affect survival of primary cortical neurons^9^. However, here we clearly show that over an extended timeframe GR50 becomes toxic to cortical and motor neurons, albeit less acutely than its longer-length GR100 counterpart. To study this chronic toxicity in vivo, we established the first GR50-expressing mouse model using a ubiquitous promoter rather than a neuronal-specific promoter, as has been previously done with larger repeat lengths^14^. Unlike in vitro, GR50-GFP expression appears predominantly diffuse and localized to the cytoplasm in expressing cells. In this model, neurons are more likely to express detectable levels of GR50-GFP than other cell types of the central nervous system. Although GR50 expression does not affect the survival of the mouse per se, it triggers significant pathological changes. Female GR50-GFP expressing mice have a reduced lower motor neuron count that is preceded by an increase in astrocyte reactivity. GR50-GFP expressing females also show an early deficit in sciatic nerve function without evidence of demyelination. These observations correlate with defects in motor performance and impaired gait dynamics. GR50-GFP females also display evidence of impaired spatial behavior and anxiety. All told, despite no reduction in survival, neurodegenerative features in line with C9orf72 ALS/FTD disease phenotypes are detected in female mice expressing GR50-GFP.

Interestingly, GR50-GFP males have a different histopathological profile than females, where no loss of lower motor neuron or significant change in astrocyte reactivity is detected. Male GR50-GFP mice also do not show evidence of impaired cognitive behavior as measured by open field, nor do they show impaired sciatic nerve function or gait abnormalities. However, GR50-GFP males have impaired rotarod performance compared to control-GFP mice. This is not the first instance where sex-dependent differences in mice attempting to recapitulate C9orf72 ALS/FTD relevant phenotypes are observed^44^. While we are not sure what is responsible for this unexpected distinction in phenotype presentation between males and females, differences in the response to GR50 expression from a hormonal or neuroinflammatory perspective could be contributing factors. Additionally, sex-dependent differences in type and severity of disease onset have been observed in ALS patients^45,46^. Because male GR50-GFP mice show a rotarod performance deficit, we hypothesize that this phenotype could rely on other neuronal populations of lumbar spinal cord that may have affected their balance, coordination, or reflexive instincts, as male GR50-GFP mice did show a slight reduction in lumbar neuron counts with age. Increased weight may also play a role in the rotarod performance of males. In addition to sex-specific differences, we also observe a temporal component to some of the identified phenotypes. For example, an early CMAP amplitude deficit is observed in GR50-GFP females compared to control-GFP females at 3 months that is lost at 6 months. At 3 months of age, we also note a deficit in time spent in the center of the cage in GR50-GFP females that is not observed with age as measured in the open field assessment. We consider the possibility that these temporally specific phenotypes may be due to a developmental delay, though this is not reflected in weight or cortical neuronal cell counts.

Our GR50-GFP mouse model displays what we consider to be a chronic phenotype. From a gross perspective, we are not able to discern genotype solely by monitoring these animals in their cages. We do not observe phenomena such as limb dragging or lack of hindlimb splay that would indicate a difference in cohorts that is potentially genotype specific. Furthermore, when performing our systematic assessment of GR50-GFP mice compared to control-GFP, we do not observe spontaneous death of any test subjects in the cohort out to 12 months of age. Additionally, observed subtle phenotypes are not progressive throughout the lifespan of the mice. For example, motor neuron loss in female GR50-GFP mice shows a deficit without exacerbation over time. The chronic consequences of GR50 expression in our animals differ from other mouse models expressing GR of greater lengths. Though not directly comparable to our model as the method of DPR expression varies from neuronal-specific knock-in^14^ to viral delivery^13,15^, mice expressing GR with repeat lengths ranging from 80 to 200 display varying but enhanced histopathological features, ranging from a GR localization pattern that may include nuclear aggregates, to reduced cortical complexity, impaired motor performance, TDP-43 pathology, and diminished survival. It is worth postulating that GR50 expression correlates with a length dependent toxicity in vivo, though direct comparison through consistent expression methods (e.g. identical promoter systems) is necessary. Another possibility is that the GFP tag alters the toxicity of GR50 in vivo, as the relative size of the GFP to the repeats increases as shorter repeat lengths are used. The field has demonstrated a difference in DPR toxicity between DPRs of identical sizes with identical fluorescence tags, as well as difference in vitro and in vivo survivability when DPRs of distinct types are expressed without tags, which lends itself to the suggestion that the GFP tag has minimal culpability in the toxicity profile observed. It has been reported that a highly expressible CAG promoter driven GR100-GFP AAV mouse model displays a toxic phenotype^15^. In this study, our GR50-GFP construct is integrated in a safe harbor site and utilizes a weaker Rosa26 promoter that expresses at more patient-relevant levels. Therefore, systematic development of GR-GFP mouse models featuring different GR lengths would need to be generated to evaluate GR length-dependent toxic phenotypes in vivo and properly address this question. Even so, the GR length window to assess the effect of the reporter tag would be quite narrow (at least between GR50 and GR100).

Because the phenotype detected in our GR50-GFP animals is subtle, we wondered why expression of GR50 does not correlate with more overt consequences. The localization pattern of GR50 may contribute to the consequences of its expression. In vitro, GR50 can be observed in both a diffuse and aggregated form, with aggregates appearing in the nucleus. This localization to the nucleus may be consequential as DPR expression has correlated with evidence of nucleolar stress. However, in human post-mortem tissue, GR detection is primarily diffuse with localization either to the cytosol or perinucleus in expressing cells^22^, in line with what was detected in our GR50-GFP mouse. That isn’t to say that nuclear aggregation in rodent model systems is not possible, as others who have developed GR mouse models with lengths of at least 80 repeats show evidence of nuclear aggregates, although not widespread. Indeed, the lack of nuclear aggregation in our model could contribute to the chronic phenotype observed. The correlation between DPR length and aggregation state is speculative, but worth investigation, especially in model systems lacking the full disease context. Additionally, this is not the first C9-associated mouse model to lack a full recapitulation of the pathological and phenotypic consequences seen in the human condition^47^. Thus, we consider the possibility of species-dependent differences in C9-related disease phenotypes.

This GR50-GFP mouse offers, however, a unique opportunity to identify methods of exacerbating a subtle phenotype. If this were the case, the expression of GR50 could be regarded as a disease modifier. Phenotype exacerbation can be elicited using a second-hit form of stress and has been demonstrated as a method of modulation in other neurodegenerative diseases^48–50^. In this case, administering an additional stressor to our GR50-GFP mice may elicit measurable phenotypic consequences in the form of increased cell loss or enhanced motor and gait deficits. It may also modulate the dynamics of GR50 itself, potentially influencing its aggregation state and localization. Second-hit stressors relevant to C9orf72 ALS/FTD include increasing neuronal activity and often rely on modulating RAN translation itself^51–53^, a mechanism on which our model does not rely. Nevertheless, the chronic consequences of GR50 in our model demonstrate C9orf72 ALS/FTD relevant phenotypes that can be manipulated for insight into the role of this DPR in overall disease pathogenesis.

## Materials and methods

### Primary cell culture and in vitro longitudinal survival assessment

All procedures involving rats were approved by the Institutional Animal Care and Use Committee at Thomas Jefferson University. Primary cortical neurons were isolated from embryonic day 16 Sprague–Dawley rats as previously described^54,55^. Cortical neurons were dissociated and plated in 24-well glass bottom culture plates coated with poly-d-lysine in neurobasal media supplemented with 1% l-glutamine, 2% B27 and 1% penicillin–streptomycin and cultured for 7 days with two half-media changes prior to transient transfection. Primary motor neurons were isolated and dissociated from embryonic day 14.5 rats as previously described^56^ with modifications. Spinal cords were dissected from the embryos and fragmented mechanically into smaller pieces. Spinal cord fragments were incubated with 0.025% trypsin and manually triturated. The spinal cord homogenate was spun through a 4% w/v bovine serum albumin (BSA) cushion to collect the dissociated cells. To specifically isolate the motor neurons, dissociated cells were spun through a 10.4% Optiprep (Sigma) cushion for 55 min without brake. The motor neuron band at the Optiprep interface was collected and spun through two 4% BSA cushions. Motor neurons were resuspended in neurobasal supplemented with B27, 0.25% l-glutamine, 0.1% beta-mercaptoethanol and 2% horse serum and plated in 24-well glass bottom culture plates coated with poly-d-lysine and laminin. Motor neurons were cultured for 5 days with two half-media changes prior to transient transfection. Transient transfection of the primary cortical and motor neurons was performed as previously described using characterized GR-GFP of various lengths (400 ng) or control-GFP plasmids (400 ng) co-transfected with td-Tomato (125 ng)^9^. Death of co-transfected GFP-td-Tomato or GR_n_-td-Tomato was defined by cell blebbing, cell bursting, or loss of the td-Tomato fluorescent signal.

### Creation of GR50 and control mice

All procedures involving mice were approved by the Institutional Animal Care and Use Committee at Thomas Jefferson University. FLAG-GR50-GFP or FLAG-GFP was integrated using a knock-in FAST cassette^23^ system at the ROSA26 locus^24,25^ under the ROSA26 promoter^25,26^. The GR50 sequence was encoded using a randomized codon strategy to allow for protein production absent of repeat-rich RNA. Animals were generated at Ingenious Targeting Laboratory (Ronkonkoma, NY, USA) on a C57BL/6 background. Successful knock-in was confirmed via PCR. Heterozygous animals were crossed with CAG-*Cre* expressing animals on a C57BL/6 background to facilitate STOP codon excision and subsequent expression of FLAG-GR50-GFP or FLAG-GFP. CAG-*Cre* mice were a gift from Dr. Yuichi Obata, Riken BioResource Center, Japan.

### Animal husbandry

All animals were housed in standard cages and provided with food and water ad libitum in a temperature, humidity, and light-controlled animal facility. Female breeders were given a 6% fat diet. All other mice used for general line maintenance and experimental assessments were given a traditional 4.5% fat diet. To minimize the possibility of cage effects in our analyses, animals were housed across multiple cages and sacrificed on a rolling schedule such that not all animals for a particular time point were sacrificed on the same day. All animal husbandry and procedures were carried out with the approval and oversight of the Thomas Jefferson University Institutional Animal Care and Use Committee and the National Institutes of Health Guide for the Care and Use of Laboratory Animals, as well as the recommendations in the ARRIVE guidelines.

### Western blot analysis

Mice were euthanized using CO_2._ Tissue samples were obtained from mice following cervical dislocation and dissection on ice. Samples not immediately used were flash frozen and stored at − 80 °C. Each tissue sample was lysed in RIPA buffer with protease inhibitor (Sigma-Aldrich, PPC1010) per sample. Samples were homogenized using a hand homogenizer. For liver, additional sonication was required. Protein amount was quantified using a Bradford Assay at a 1:5 dilution. 20 µg of protein from each sample were boiled for 10 min in loading buffer. Samples were loaded onto stain-free mini gels (Bio-Rad) and run at 75 V for approx. 2 h. Gels were transferred to a 0.2 µm nitrocellulose membrane using the Bio-Rad Trans-Blot Turbo at 1.3 amps, 25 V for 7 min. Gels were imaged before transfer using the Chemi-doc stain-free feature (Bio-Rad) to ensure equal loading. Membranes were also imaged using the stain-free feature post-transfer to ensure efficient and equal protein transfer. Membranes were blocked in 5% milk in tris-buffered saline solution with tween (TBST) for one hour at room temperature with rapid shaking. Primary antibody (GR, Rt, Millipore MABN778, 1:5000 or GFP, Rb, Proteintech 50430-2-AP, 1:3000) was diluted in 5% BSA in TBST and incubated at 4 °C with gentle shaking. Following primary incubation, membranes were rinsed with TBST 4 × for 15 min each with rapid shaking and then incubated with secondary HRP (Gt, Thermo Fisher, 18-4818-82, 1:5000 or Rb, Thermo Fisher, NA9340V, 1:5000) in 5% milk in TBST for 1 h at room temperature with gentle shaking, followed by 2 × rinses with TBST for 15 min each with rapid shaking. To develop, membranes were covered in ECL (GE Healthcare, 17030484) for 2 min and imaged using chemiluminescence on the Chemi-doc imaging system.

In addition to using the stain-free feature to ensure equal loading, beta-tubulin (Primary: Ms, Millipore 05-661, 1:5000; Secondary HRP: Ms, Cytiva, NA9310, 1:5000) was used as a loading control. For blots in which multiple proteins could be detected with adequate separation, specifically GFP (~ 25 kDa) and beta-tubulin (~ 55 kDa), membranes were cut at ~ 37 kDa post-transfer, prior to blocking and antibody hybridizations. For blots in which multiple proteins could be detected from a single membrane, membranes were stripped between hybridizations with a stripping buffer consisting of 20 mL 10% SDS and 12.5 mL of Tris HCl, pH 6.5, with 0.8% beta-mercaptoethanol added immediately prior to stripping. Membranes were placed in stripping buffer and kept at 37 °C for 15 min, followed by a 10 min rapid shaking at room temperature. Membranes were moved to TBST and rinsed 2 × for 15 min each. Membranes were re-imaged to ensure adequate stripping before re-blocking. Primary and secondary incubations were repeated as described. Band intensity was quantified using the Image Lab software (Bio-Rad).

### Tissue preparation for histological analyses

Mice were anesthetized and perfused by transcardial puncture with PBS and chilled 4% paraformaldehyde (PFA). Relevant specified tissues were immediately dissected and post-fixed in 4% PFA for 24 h, followed by a 24-h incubation in phosphate buffer saline (PBS). For cryoprotection, samples were moved to a 30% sucrose solution for a minimum of 48 h until the sample no longer floated. Samples were embedded in OCT compound (Sakura, 4583) embedding medium and frozen. Cortices were sectioned serially in the coronal orientation while spinal cord was sectioned serially in the transverse orientation and collected on positively charged glass slides. Cortex, spinal cord, and liver were sectioned at a thickness of 30 µm. Slides were kept at − 20 °C until analysis. To collect sections approximately containing motor cortex, the connected corpus callosum and the anterior hippocampus were used as anterior and posterior landmarks, with sections between these landmarks used for analysis.

### General immunofluorescence

Frozen slides were warmed for 30 min on a slide warmer to remove excess OCT medium. Slides were washed 3 × with PBS and blocked with 5% normal serum and 0.3% Triton X-100 (Sigma, X100) in PBS for 1 h. Slides were incubated with primary antibody in an antibody dilution buffer consisting of 0.3% Triton X-100 and 1% (w/v) BSA. Primary antibodies used: GFP (Chk, Millipore, AB16901, 1:2500); NeuN (Rb, Cell Signaling Technology, 24307S, 1:1000); iba1 (Rb, Wako Chemicals, 019-19741, 1:1000); s100β (Rb, Abcam, ab41548, 1:1000); cleaved caspase-3 (Rb, Cell Signaling Technology, 9661S, 1:400); TDP-43 (Rb, Cell Signaling Technology, 89789S, 1:400); GFAP (Ms, Cell Signaling Technology, 3670S, 1:300). Slides were rinsed 3 × with PBS and incubated with fluorescent secondary in antibody dilution buffer at a 1:500 dilution. Secondary antibodies used: Alexa Fluor 488 goat-anti-chicken (Thermo Fisher, A-11039); Alexa Fluor 594 donkey-anti-rabbit (Invitrogen, A21207); Alexa Fluor 546 donkey-anti-mouse (Life Technologies, A10036). Slides were rinsed 3 × protected from light and mounted with Vectashield antifade mounting medium with DAPI (Vector Laboratories, H-1200).

### ChAT immunofluorescence

Frozen slides containing sections from both rostral and caudal spinal cord were warmed for 30 min on a slide warmer to remove excess OCT medium. Slides were washed 3 × with PBS and incubated with 0.4% Triton X-100 in PBS for 30 min. Slides were rinsed 3 × and incubated with 5% BSA for 1 h. Slides were rinsed 3 × with PBS. ChAT primary antibody (Gt, Millipore, AB144, 1:1000) was diluted in 0.1% Triton X-100, 3% fetal bovine serum in PBS for 48 h at 4 °C. Slides were rinsed 5 × for 15 min each with PBS. Secondary Alexa Fluor 647 donkey-anti-goat (1:500, Invitrogen, A21447) was diluted in 0.1% Triton X-100, 3% fetal bovine serum in PBS for 2 h at room temperature, followed by 5 × rinsing for 15 min each with PBS. Slides were incubated with Hoechst (Thermo Fisher, 33,258 1:5000) in PBS for 15 min, rinsed 3 × with PBS, and mounted. Quantification was performed on a minimum of 6 fields per animal in an automated manner using NIS Elements (Nikon). Images were thresholded for intensity and cell size, allowing for a blinded count of cells.

### Hindlimb neuromuscular junction immunofluorescence

Hindlimb NMJ morphology was visualized as previously described^57^ with modifications. Animals were euthanized with CO_2_ with a secondary cervical dislocation and kept on ice. Whole hindlimb was dissected above the knee joint. The entirety of the soleus and extensor digitorum longus muscles were dissected and postfixed in 4% PFA for 20 min. Muscles were subsequently rinsed 3 × for 10 min each with PBS pH 7.4, followed by a 30-min incubation in 0.1 M glycine. All subsequent steps were performed with light-prevention. To visualize postsynaptic acetylcholine receptors, muscles were incubated with alpha bungarotoxin, Alexa Fluor 594 conjugate (Thermo Fisher, B13423, 1:200) for 15 min, then rinsed 3 × for 10 min each with shaking with PBS pH 7.4. The muscles were then permeabilized with 100% cold methanol for 5 min at − 20 °C, rinsed 3 × with PBS pH 7.4, and blocked with 0.2% Triton X-100 in 2% BSA in PBS with gentle shaking. To visualize the presynaptic regions, the muscles were incubated in a primary antibody cocktail containing SMI312 (BioLegend, 837904, 1:1000) and SV2 (DSHB, SV2-s, 1:10) in 0.2% Triton X-100, 2% BSA in PBS overnight at 4 °C with gentle shaking. Muscle was rinsed 3 × with 0.2% Triton X-100 in 2% BSA in PBS and incubated with secondary FITC AffiniPure goat-anti-mouse IgG, FCγ subclass 1 specific (Jackson ImmunoResearch, 115-095-205, 1:200) in 0.2% Triton X-100 in 2% BSA in PBS for 1 h at room temperature with gentle shaking. Tendons were removed and muscles were mounted using Vectashield antifade mounting medium with DAPI (Vector Laboratories, H-1200). Slides were kept under a weight for 24 h prior to imaging.

### Liver immunohistochemistry

30 µm liver sections underwent a periodic acid Schiff (PAS) staining according to manufacturer specifications (Abcam, ab150680).

### Sciatic nerve CMAP

Electrophysiology recordings were carried out as previously described^58^. Briefly, CMAPs were recorded using the Neurosoft-Evidence 3102evo electromyograph system (Schreiber and Tholen Medizintechnik GmbH, Stade, Germany). All recordings were collected at room temperature with the experimenter blinded to the mouse genotypes. Mice were anesthetized with 2–3% isoflurane for testing. Needle electrodes (27G Stainless steel, Technomed Medical Accessories, The Netherlands) were placed at the sciatic notch for proximal stimulation, at the Achilles tendon for distal stimulation, and in the lateral plantar muscles for recording. Stimulation voltage was optimized for maximal response and testing completed by stimulating with very short electrical impulses (< 0.2 ms). Amplitude was calculated peak to peak. Latency was calculated from stimulus onset. Nerve conduction velocity was calculated as the difference between the proximal and distal stimulation sites, divided by the difference between the corresponding latencies.

### Rotarod assessment

Mice received two training sessions prior to testing on day 3. For the first day of training, animals were exposed to 3 10-min sessions on the rotarod at a speed of 4 rpm with a minimum of 10-min breaks in-between sessions. Day 2 of training consisted of three 10-min sessions using a 4–40 rpm acceleration with a minimum of 10-min breaks in-between sessions. The parameters of the second day of training were used for the testing session on day 3. Latency to fall was recorded as the difference between the time stamp at which the animal fell from the bar and the time stamp of the start of acceleration. The average of the three latency measurements per animal was used for analysis. All test sessions were performed in the light cycle phase (10 a.m.–5 p.m.).

### Grip strength

A grip strength meter was used to measure hind limb grip strength (DFIS-2 Series Digital Force Gauge, Columbus Instruments, OH). The hind limbs grasped the bar while the peak pull force recorded by the force transducer was measured in Newtons. One experimenter, blind to the experimental groups, was used for the data presented to control for the speed of the tail pull. Mouse fore paws were restricted from the measurement by allowing mice to grip their fore paws to a rod held by the experimenter. Three recordings per mouse were taken. Recordings in which the hind paws were not on the bar in parallel, all digits were not grasping the bar, or one paw released prior to pull, were not used. All test sessions were performed in the light cycle phase (10 a.m.–5 p.m.).

### Open field assessment

Animals were acclimated to the behavioral assessment room for 15 min prior to assessment. Mice were individually placed in an open-top chamber with a roaming area of approximately 40 × 40 cm^2^. Animals were placed in the center of the chamber and individually recorded for 10 min using a ceiling-mounted camera as they explored freely. Footage was analyzed using the ANY-maze video tracking software (Stoelting Co., Illinois, USA). The experimenter was blind to the genotype of the mouse during assessment. All test sessions were performed in the light cycle phase (10 a.m.–5 p.m.).

### Quantitative gait analysis

Gait assessment was performed using the DigiGait quantitative gait analysis platform (Mouse Specifics, Massachusetts, USA). Mice ran on a clear treadmill enclosed with a polycarbonate chamber at a speed of 15 cm/s while the ventral surfaces of all four paws were recorded. Animals were allowed to habituate to the chamber briefly before running. Recordings of approximately 10 s per mouse were acquired to provide enough steps for analysis. All test sessions were performed in the light cycle phase (10 a.m.–5 p.m.). Footage was analyzed using the associated DigiGait analysis software and used the digitized area of the paw on the treadmill to quantify the indices measured. The entirety of the data output relevant to animal gait was used in the principal component analysis, which was performed using MATLAB and RStudio.

### Quantifications and statistical analyses

Data collection was performed blinded to genotype when indicated. Automated cell counts and intensity measurements were performed using the NIS Elements Software (Nikon, New York, USA) or ImageJ (National Institutes of Health). Hindlimb gait principal component analysis was performed using MATLAB (MathWorks, Massachusetts, USA) and RStudio (Massachusetts, USA). Statistical analysis was performed in GraphPad Prism (GraphPad, California, USA). Statistical assessments include Kaplan–Meier survival assessment with log-rank testing, multiple unpaired *t* tests, one-way analysis of variance (ANOVA) with Tukey’s multiple comparisons test, or two-way ANOVA with Sidak’s multiple comparisons test. Data is reported as mean ± s.e.m.; p < 0.05 was considered statistically significant. Animal sample sizes for males and females were determined through both a-priori sample size calculation and published data suggesting comparable minimum sample sizes for similar behavior assessments^14,59^.

## Supplementary Information

Supplementary Figures.

Supplementary Information.

## Data Availability

The datasets used and/or analyzed in the current study are available from the corresponding author upon reasonable request.
