# Supplementary material for: A mouse model with widespread expression of the C9orf72-linked glycine–arginine dipeptide displays non-lethal ALS/FTD-like phenotypes
Source: Sci Rep. 2022 Apr 4;12:5644. doi: 10.1038/s41598-022-09593-z (PMC8979946; doi:10.1038/s41598-022-09593-z)
Supplement: Supplementary file 1 — Supplementary Figures. [file 41598_2022_9593_MOESM1_ESM.pdf]

Supplemental Figure 1

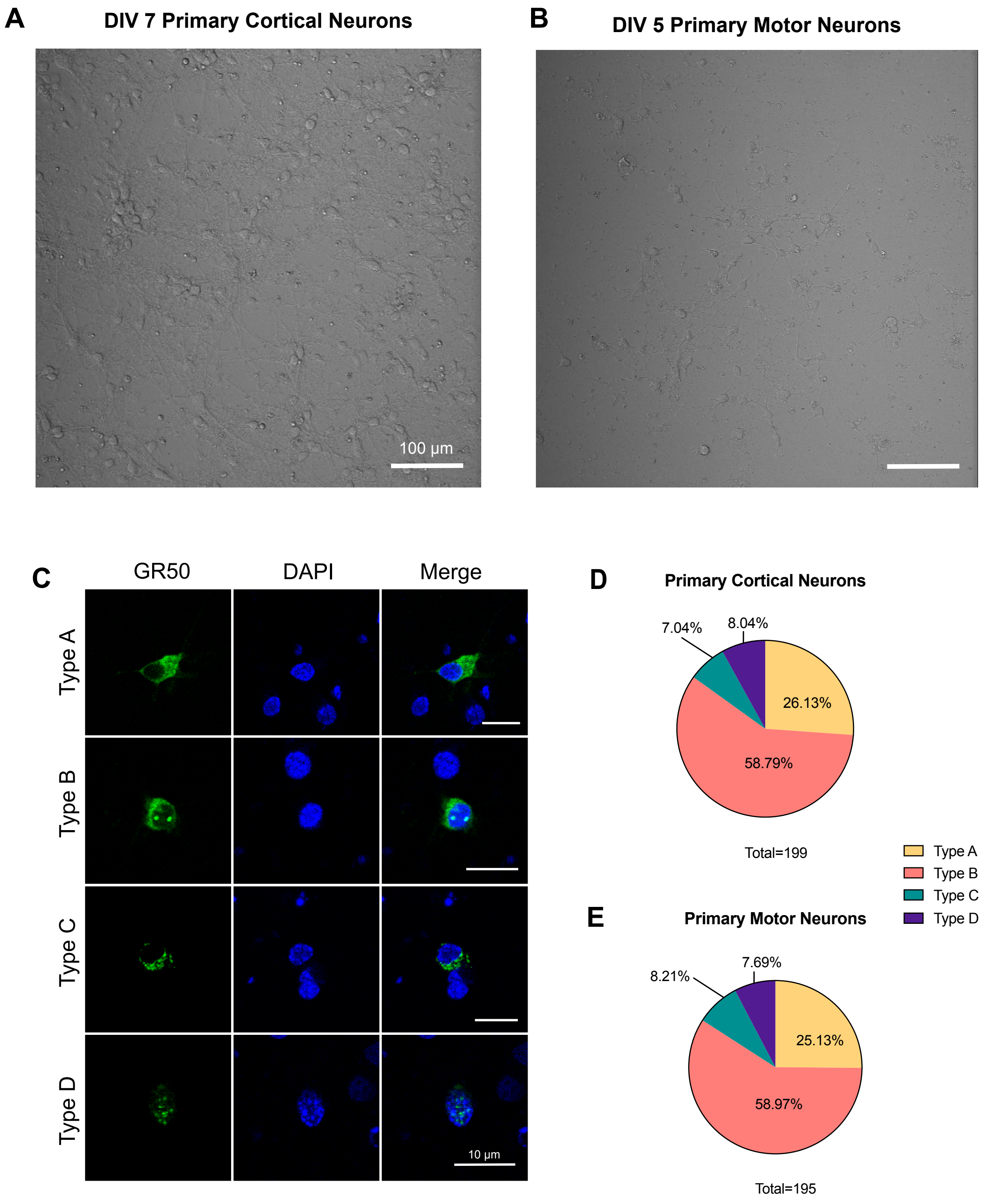

Supplementary Fig. 1 in vitro assessment of GR50 localization patterns. A) Representative brightfield image of day 7 primary cortical neurons. Scale bar=100 µm. B) Representative brightfield image of day 5 primary cortical neurons. Scale bar=100 µm. C) GR50 localization patterns in transiently transfected neurons 24 hours after transfection. Type A: diffuse, cytosolic. Type B: Cytosolic with evidence of nuclear aggregates. Type C: cytosolic aggregates only. Type D: Nuclear aggregates only. Scale bar=10µm. Percent distribution of localization type quantified in primary cortical neurons (D) and primary motor neurons (E). m=199 primary cortical neurons, 195 primary motor neurons.

Supplemental Figure 2

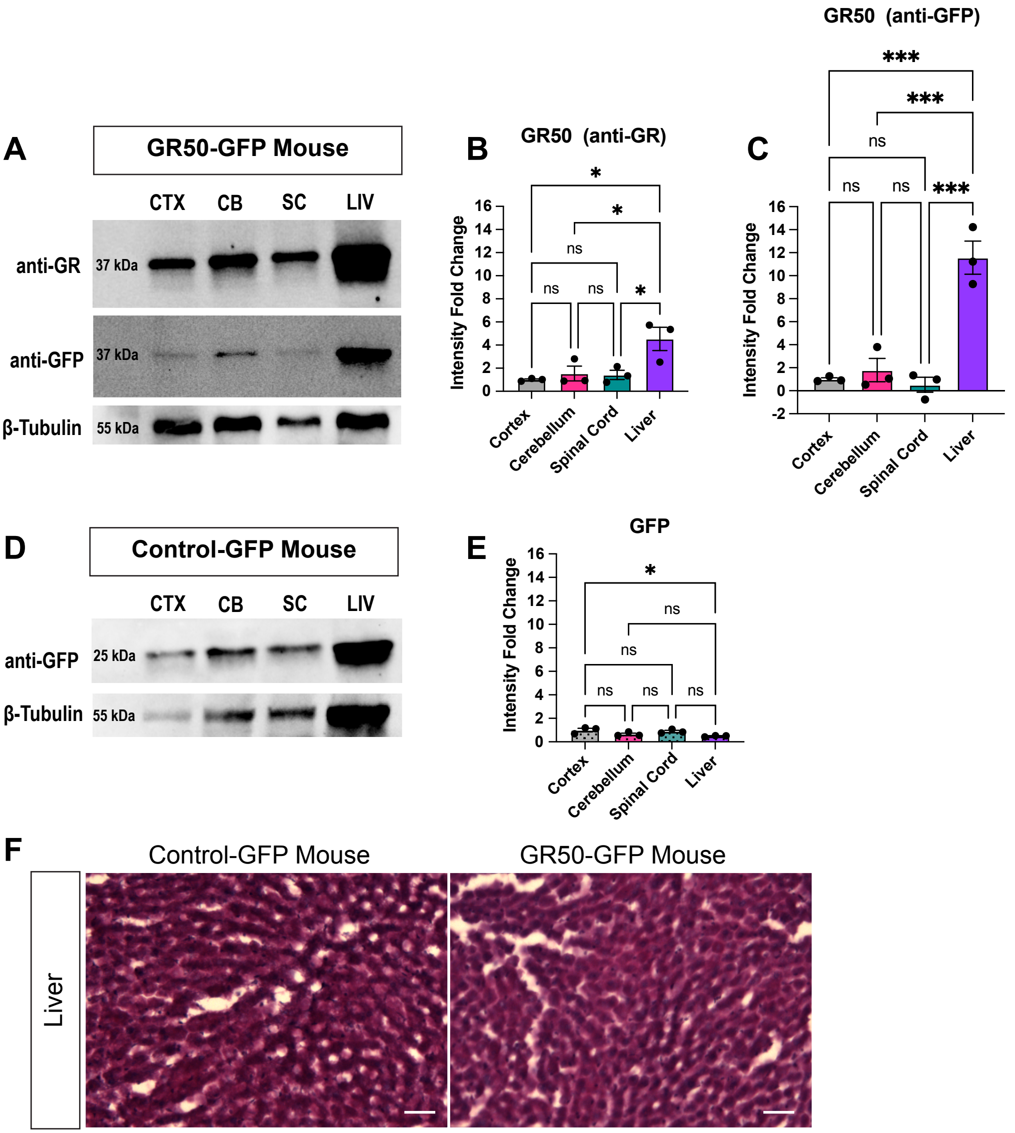

Supplementary Fig. 2 GR50 expression under a ubiquitous promoter facilitates expression across CNS and non-CNS tissues. A) Representative western blots of GR and GFP expression in cortex (CTX), cerebellum (CB), spinal cord (SC), and liver (LIV) of a 3-month-old GR50-GFP mouse. B) Quantification of change in protein expression across tissues of GR50-GFP mice when probed for GR, normalized to beta-tubulin. n=3 mice, mean s.e.m. One-way ANOVA, Tukey's multiple comparisons test. Anti-GR: cortex vs. cerebellum p=0.9262; cortex vs. spinal cord p=0.9628; cortex vs. liver p=0.0171; cerebellum vs. spinal cord p=0.9990; cerebellum vs. liver p=0.0395; spinal cord vs. liver p=0.0327. C) Quantification of change in protein expression across tissues of GR50-GFP mice when probed for GFP, normalized to beta-tubulin. n=3 mice, mean s.e.m. One-way ANOVA, Tukey's multiple comparisons test. Anti-GFP: cortex vs. cerebellum p=0.9305; cortex vs. spinal cord p=0.9826; cortex vs. liver p=0.0002; cerebellum vs. spinal cord p=0.7760; cerebellum vs. liver p=0.0004; spinal cord vs. liver p=0.0002. D) Representative western blots of GFP expression in cortex (CTX), cerebellum (CB), spinal cord (SC), and liver (LIV) of a 3-month-old control-GFP mouse. E) Quantification of change in protein expression across tissues of control-GFP mice when probed for GFP, normalized to beta-tubulin. n=3 mice, mean s.e.m. One-way ANOVA, Tukey's multiple comparisons test. Anti-GFP: cortex vs. cerebellum p= 0.1606; cortex vs. spinal cord p=0.8713; cortex vs. liver p=0.0363; cerebellum vs. spinal cord p=0.4229; cerebellum vs. liver p=0.7219; spinal cord vs. liver p=0.1054. F) Periodic acid-Schiff staining of liver from a 3-month-old control-GFP and GR50-GFP mouse. Scale bar=100 μm.

Supplemental Figure 3

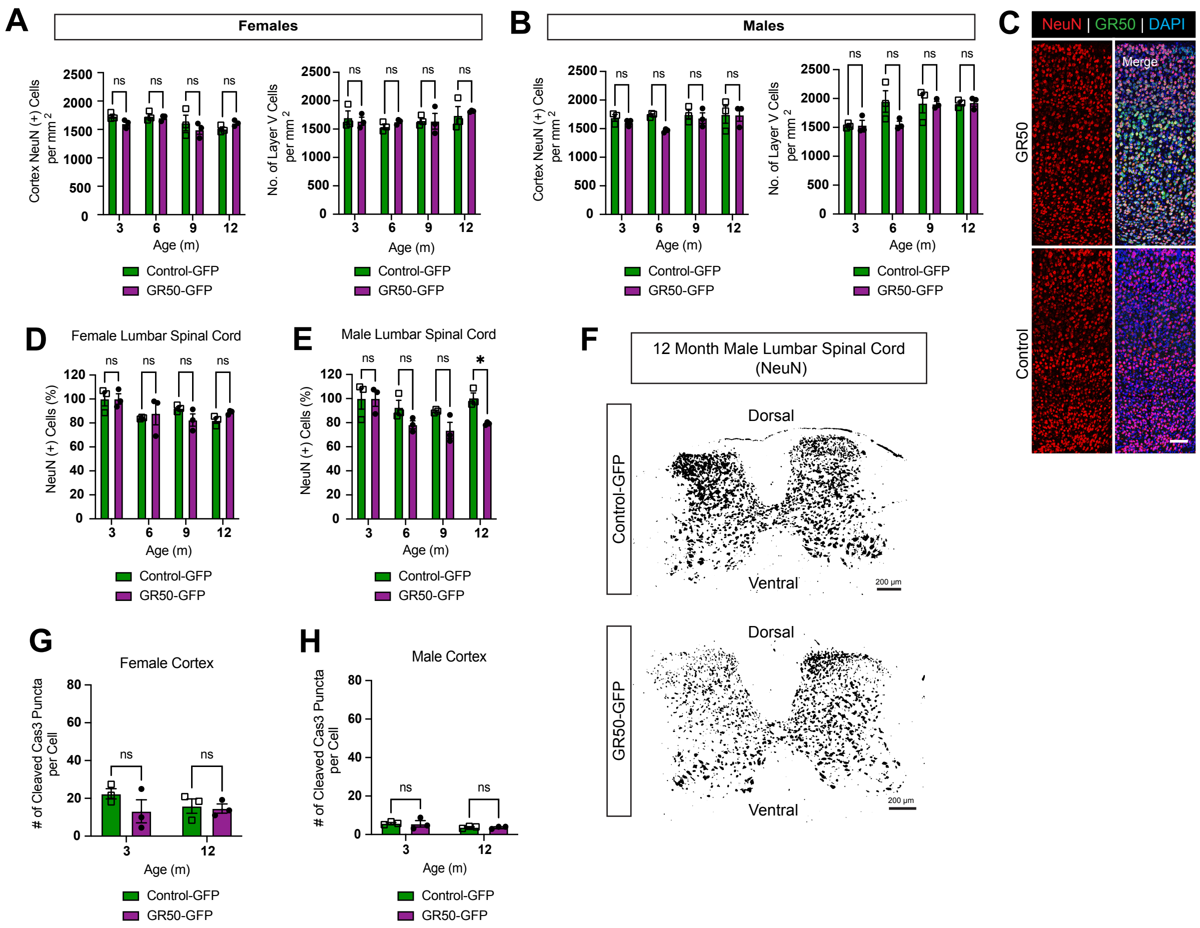

Supplementary Fig. 3 Additional histological assessments of control-GFP and GR50-GFP mice. A) Quantification of NeuN-positive cells throughout layers I-VI of cortex (left) and layer V of cortex (right) per mm2 in female mice at 3-, 6-, 9- and 12-months. n=3 mice per genotype and time-point, mean s.e.m. All layers: 3-months p=0.5257, 6-months p=0.9971, 9-months p=0.5968, 12-months p=0.7219. Layer V: 3-months p=0.9839, 6-months p=0.9536, 9-months p>0.9999, 12-months p=0.9693. B) Quantification of neun-positive cells throughout layers I-VI of cortex (left) and layer V of cortex (right) per mm2 in male mice at 3-, 6-, 9- and 12-months. n=3 mice per genotype and time-point, mean s.e.m. All layers: 3-months p= 0.9249, 6-months p= 0.1243, 9-months p=0.9773, 12-months p >0.9999. Layer V: 3-months p>0.9999, 6-months p=0.0507, 9-months p>0.9999, 12-months p >0.9999. C) Representative image of cortical layers I-VI of control-GFP and GR50-GFP mice at 3-months-old. Scale bar=100 µm. D) Quantification of percentage of NeuN positive cells in the lumbar spinal cord of female mice relative to 3 months. n=3 mice per genotype and time-point, mean s.e.m. 3-months p>0.9999, 6-months p=0.9685, 9-months p=0.5124, 12-months p=0.7844. E) Quantification of percentage of NeuN positive cells in the lumbar spinal cord of male mice relative to 3 months. n=3 mice per genotype and time-point, mean s.e.m. 3-months p>0.9999, 6-months p=0.2581, 9-months p=0.1852, 12-months p=0.0475. F) Representative binary image of NeuN-positive cells of the lumbar spinal cord at 12 months in control-GFP and GR50-GFP mice at 3-months-old. Scale bar=200 µm. G) Quantification of cleaved caspase-3 puncta accumulation within cleaved caspase-3 positive cells in the cortex of female control-GFP and GR50-GFP mice at 3- and 12-months-old. n=3 mice per time-point, min 6 fields per mouse for quantification, mean s.e.m. 3-months p=0.2666, 12-months p=0.9696. H) Quantification of cleaved caspase-3 puncta accumulation within cleaved caspase-3 positive cells in the cortex of male control-GFP and GR50-GFP mice at 3- and 12-months-old. n=3 mice per time-point, min 6 fields per mouse for quantification, mean s.e.m. 3-months p= 0.9721, 12-months p=0.9916. For all datasets, Two-way ANOVA with Sidak's multiple comparisons test was used for statistical analysis.

Supplemental Figure 4

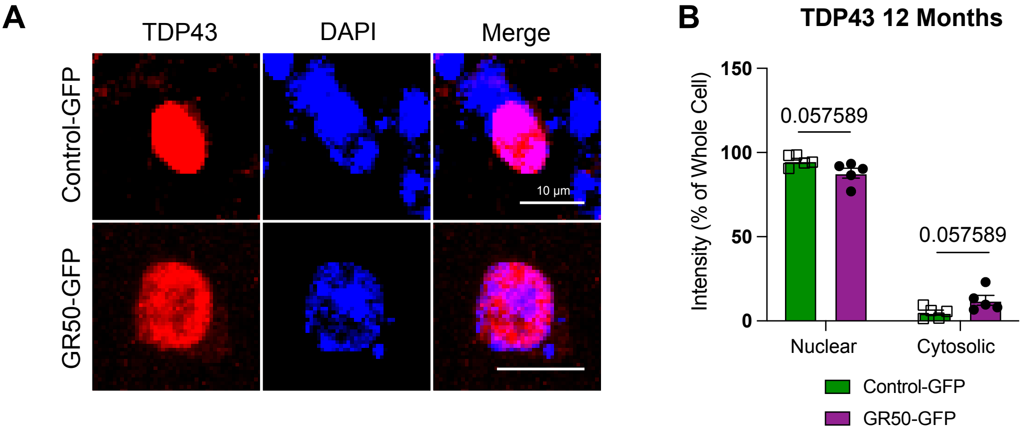

Supplementary Fig 4 TDP-43 is not mis-localized in the CNS of GR50-GFP mice. A) Representative image of TDP-43 expression in control-GFP and GR50-GFP mice at 12-months-old. Scale bar=10μm. B) Quantification of percentage of TDP-43 signal intensity in the nucleus and cytoplasm. n=5 mice per genotype, mean s.e.m, multiple unpaired t-tests. Nuclear p=0.057589. Cytosolic p=0.057589.

Supplemental Figure 5

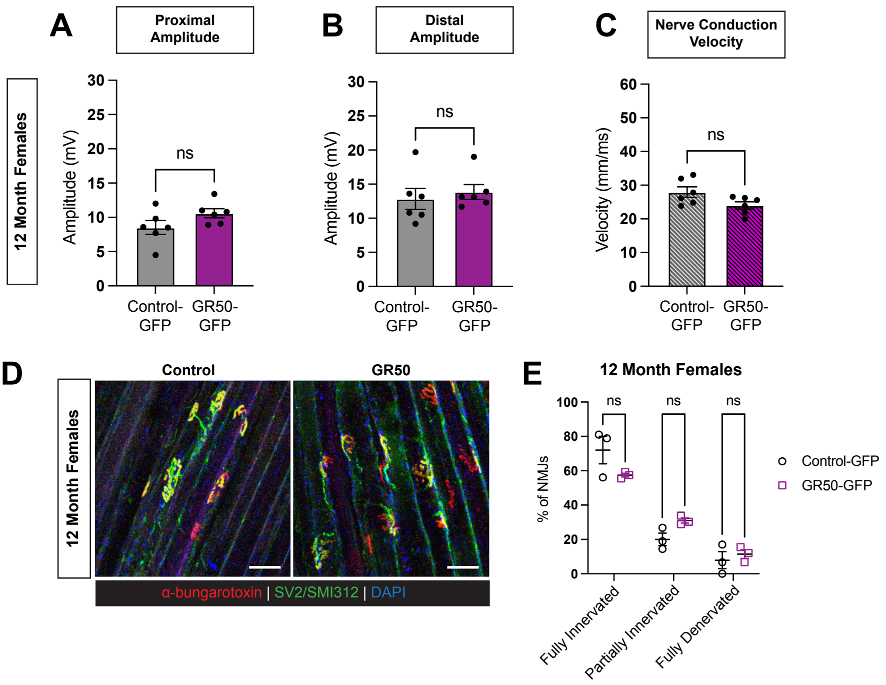

Supplementary Fig 5 12-month female CMAP and NMJ histology. A) Proximal amplitude recordings in female control-GFP and GR50-GFP mice at 12-months-old. n=6 mice per genotype; mean s.e.m, unpaired t-test, p= 0.1154. B) Distal amplitude recordings in female control-GFP and GR50-GFP mice at 12-months-old. n=6 mice per genotype; mean s.e.m, unpaired t-test, p= 0.5993. C) Nerve conduction velocity in female control-GFP and GR50-GFP mice at 12-months-old. n=6 mice per genotype; mean s.e.m, unpaired t-test, p= 0.0644. D) Representative images of the neuromuscular junctions of the extensor digitorum longus muscle of female control-GFP and GR50-GFP mice at 12-months-old stained for presynaptic SV2 and postsynaptic alpha-bungarotoxin. Scale bar=50 µm. E) Quantification of the innervation status of NMJs of the soleus and extensor digitorum longus muscles of female control-GFP and GR50-GFP mice at 12-months-old. n=3 mice per time-point, mean s.e.m. Two-way ANOVA, Sidak's multiple comparisons test. Fully Innervated: p=0.5043, Partially Innervated: p=0.2159, Fully Denervated: p=0.9195.
