# Supplementary material for: A mouse model with widespread expression of the C9orf72-linked glycine–arginine dipeptide displays non-lethal ALS/FTD-like phenotypes
Source: Sci Rep. 2022 Apr 4;12:5644. doi: 10.1038/s41598-022-09593-z (PMC8979946; doi:10.1038/s41598-022-09593-z)
Supplement: Supplementary file 2 — Supplementary Information. [file 41598_2022_9593_MOESM2_ESM.docx]

Western blots included in Verdone et al.

**Figure 2B anti-GR:**

Chemiluminescence:

1 2 3 4 5 6 7 8 9 10


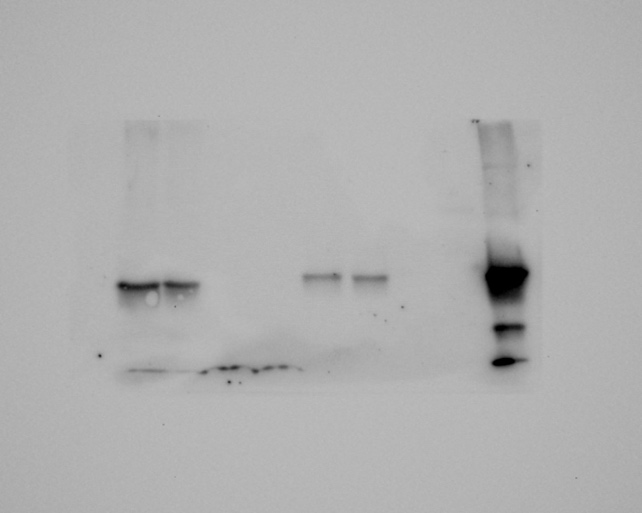


(kDa)

250

150

50

37

25


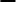

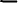

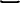

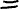


Lanes:

1. Ladder
2. Cortex GR mouse 1
3. Cortex GR mouse 2
4. Cortex GFP mouse 1
5. Cortex GFP mouse 2
6. Spinal Cord mouse 1
7. Spinal Cord mouse 2
8. Spinal Cord mouse 1
9. Spinal Cord mouse 2
10. HEK Cell lysate transfected with GR50-GFP

Notes:

- Ladders traced for emphasis.

**Figure 2B anti-GFP**

Colorimetric: Chemiluminescence:

1 2 3 4 5 6 7 8 9 10

1 2 3 4 5 6 7 8 9 10

(kDa)

250

150

75

50

37

25

20

15


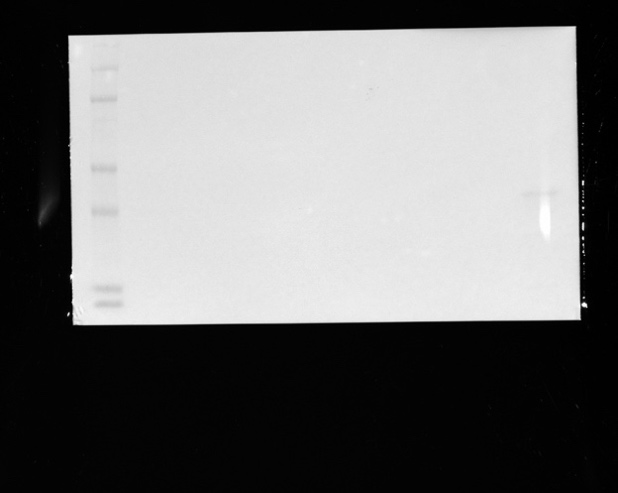

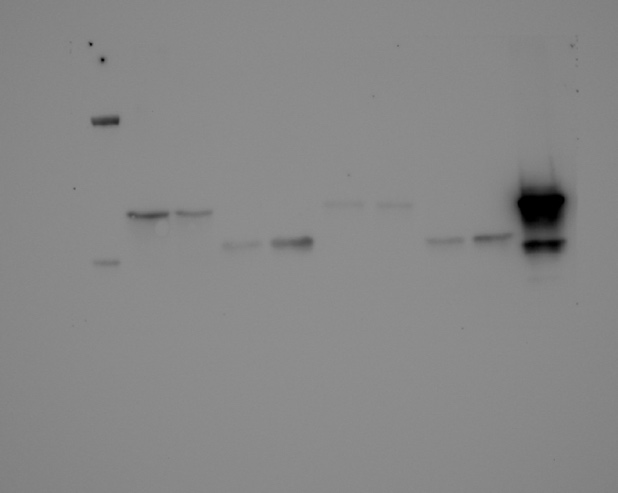


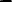

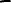

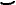

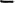

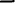

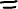


Lanes:

1. Ladder
2. Cortex GR mouse 1
3. Cortex GR mouse 2
4. Cortex GFP mouse 1
5. Cortex GFP mouse 2
6. Spinal Cord GR mouse 1
7. Spinal Cord GR mouse 2
8. Spinal Cord GFP mouse 1
9. Spinal Cord GFP mouse 2
10. HEK Cell lysate transfected with GR50-GFP

Notes:

- This membrane is the same whole membrane as shown in Figure 2B anti-GR. This blot was stripped prior to re-incubation with GFP.
- The membrane shows GFP at its molecular weight of ~25 kDa in mice expressing GFP (lanes 4, 5, 8, 9) but not GR50-GFP (lanes 2, 3, 6, 7), **confirming specific detection of GFP on an uncut membrane.**
- Ladders traced for emphasis.

**Supplementary Figure 2A: GR50 mouse; anti-GR**

Chemiluminescence:

1 2 3 4 5

**
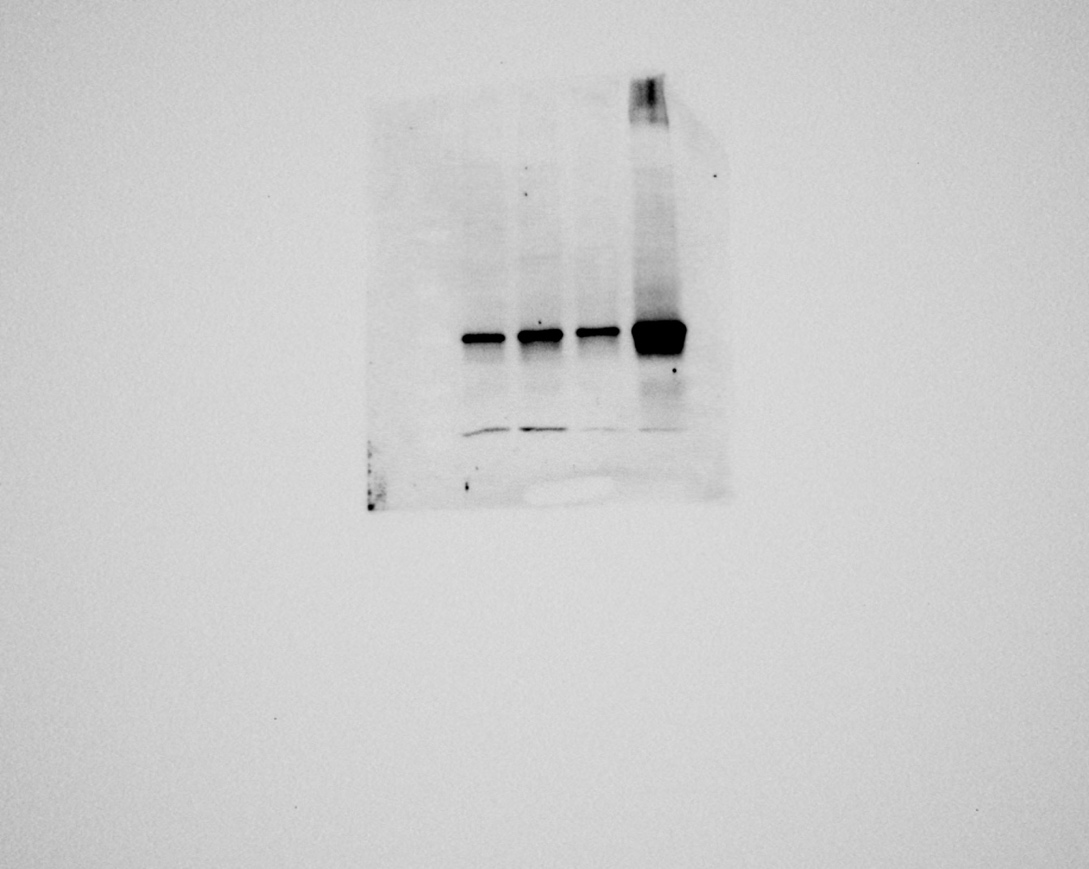
**

(kDa)

75

50

37

25


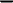

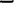

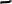

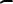


Lanes:

1. Ladder
2. GR mouse cortex
3. GR mouse cerebellum
4. GR mouse spinal cord
5. GR mouse liver

Notes:

- Ladders traced for emphasis.

**Supplementary Figure 2A: GR50 mouse; anti-GFP**

Chemiluminescence:

1 2 3 4 5

**
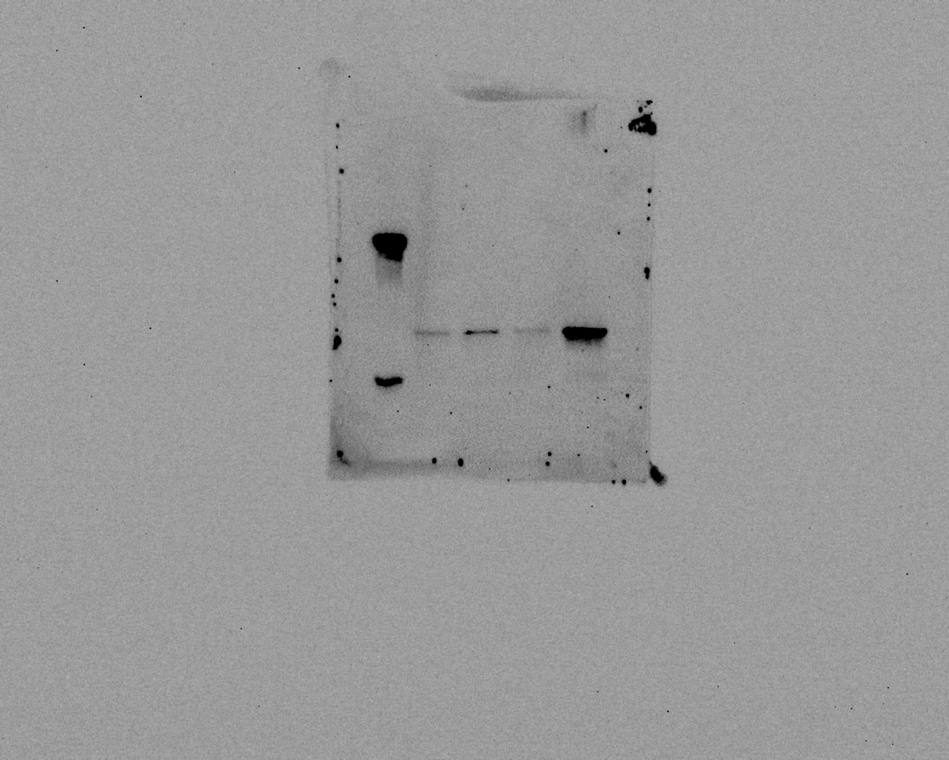
**

(kDa)

75

37

25

Lanes:

1. Ladder
2. GR mouse cortex
3. GR mouse cerebellum
4. GR mouse spinal cord
5. GR mouse liver

Notes:

- This membrane is the same whole membrane as shown in Supplementary Figure 2A anti-GR. This blot was stripped prior to re-incubation with GFP.

**Supplementary Figure 2A: GR50 mouse; anti-beta tubulin**

Chemiluminescence:

**
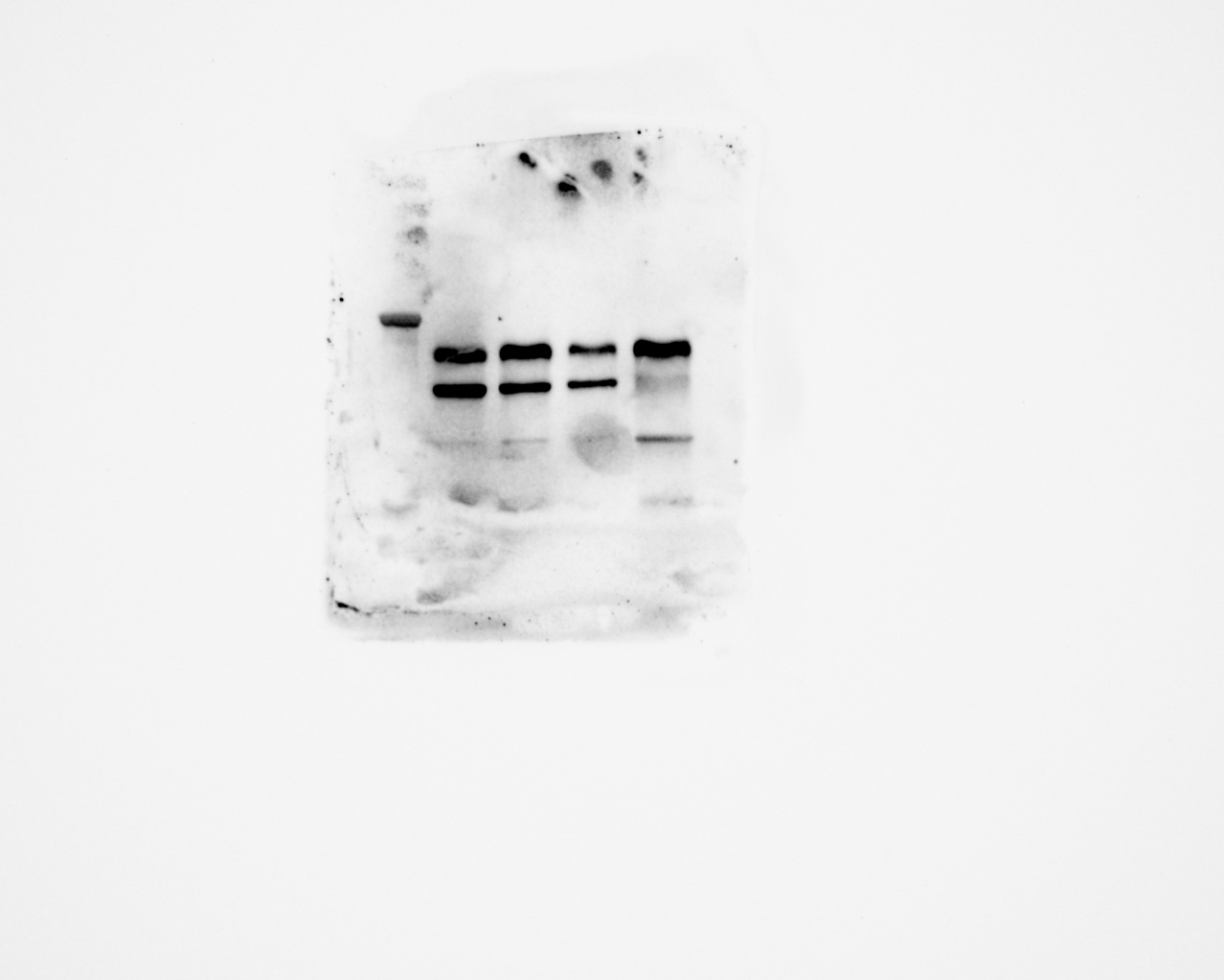
**

1 2 3 4 5

(kDa)

75

37

Lanes:

1. Ladder
2. GR mouse cortex
3. GR mouse cerebellum
4. GR mouse spinal cord
5. GR mouse liver

Notes:

- This membrane is the same whole membrane as shown in Supplementary Figure 2A anti-GR and Supplementary Figure 2A anti-GFP. This blot was stripped prior to re-incubation with beta-tubulin.

**Supplementary Figure 2D: GFP mouse; anti-GFP**

Chemiluminescence:

**
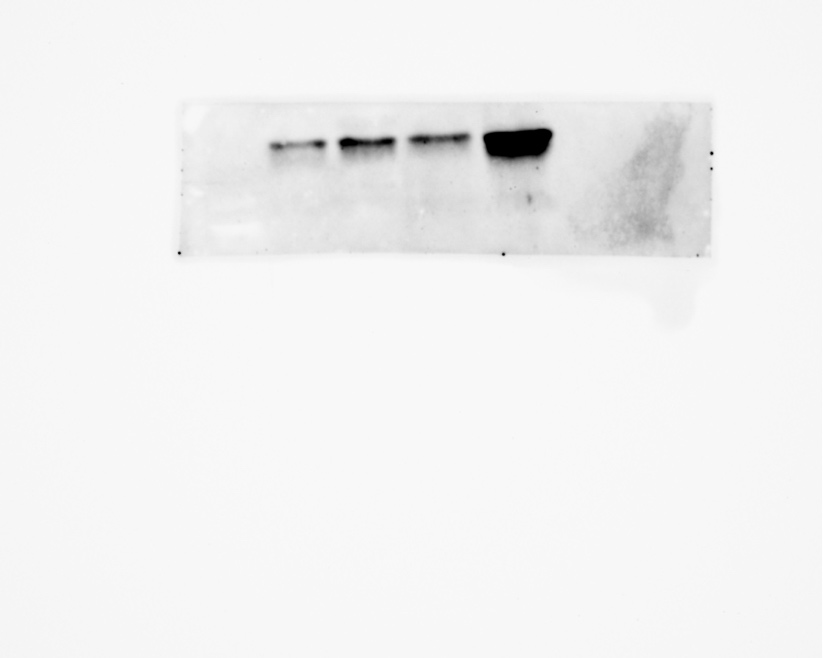
**

(kDa)

Cut at 37

20

15


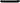

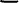


1 2 3 4 5

Lanes:

1. Ladder
2. GFP mouse cortex
3. GFP mouse cerebellum
4. GFP mouse spinal cord
5. GFP mouse liver

Notes:

- This membrane was cut at ~37 kDa prior to blocking and antibody hybridization.
- Ladders traced for emphasis.

**Supplementary Figure 2D: GFP mouse; anti-beta tubulin**

Chemiluminescence:

**
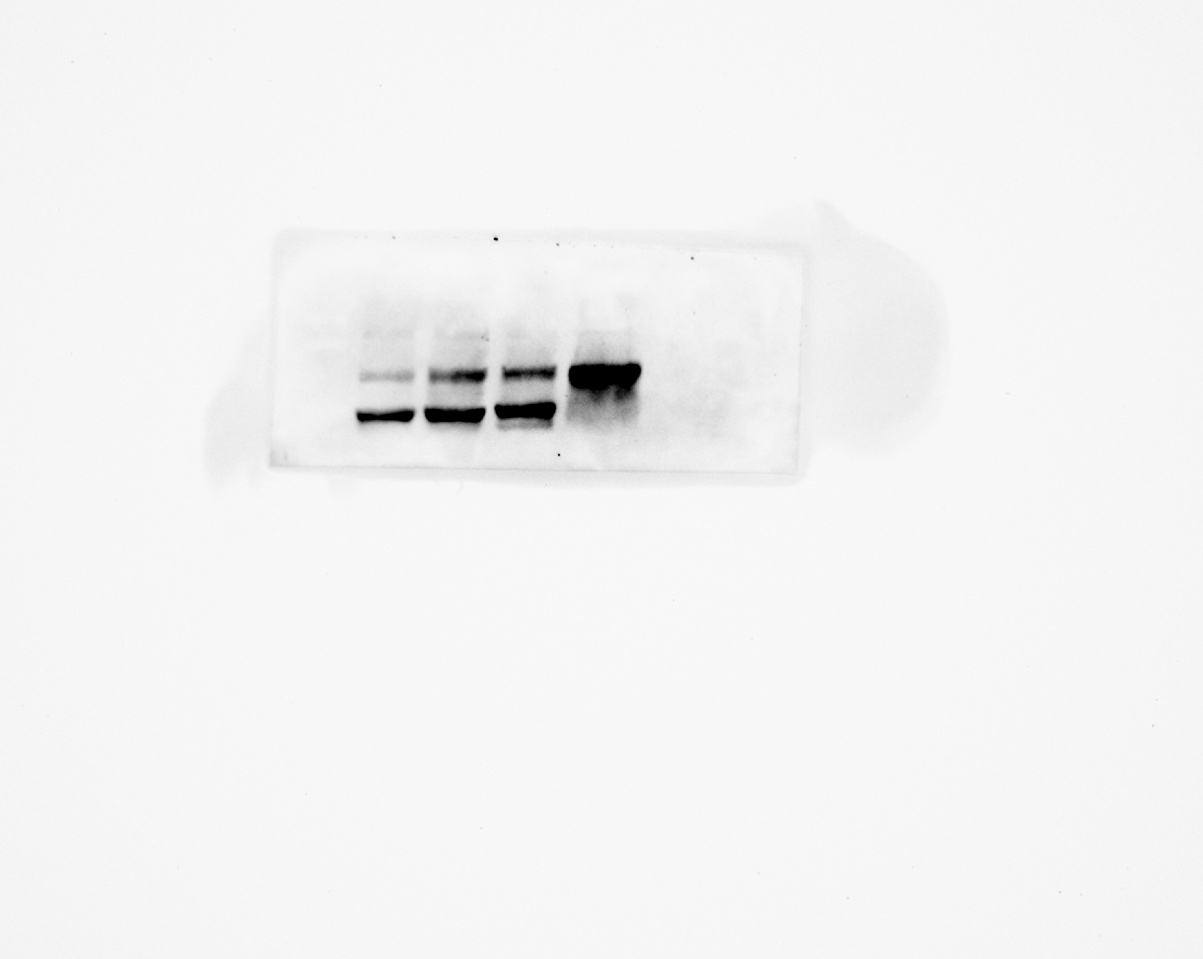
**

(kDa)

75

100

Cut at 37


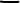

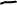


1 2 3 4 5

Lanes:

1. Ladder
2. GFP mouse cortex
3. GFP mouse cerebellum
4. GFP mouse spinal cord
5. GFP mouse liver

Notes:

- This membrane was cut at ~37 kDa prior to blocking and antibody hybridization and is the top half of the membrane shown in Supplementary Figure 2D, anti-GFP.
- Ladders traced for emphasis.

**Additional Replications of Cut Blots**

Anti- beta tubulin

1 2 3 4 5 6 7 8 9 10


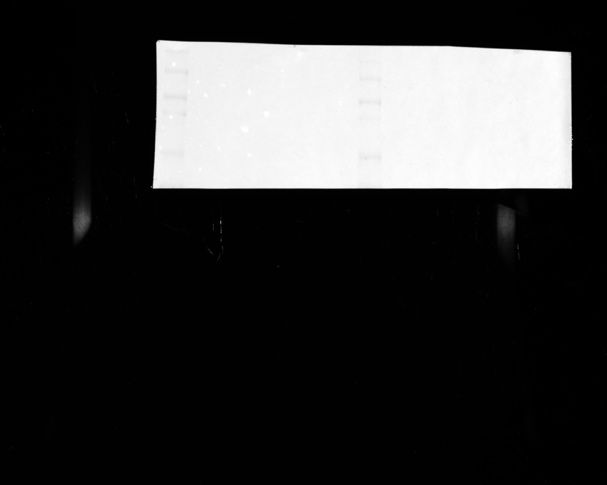

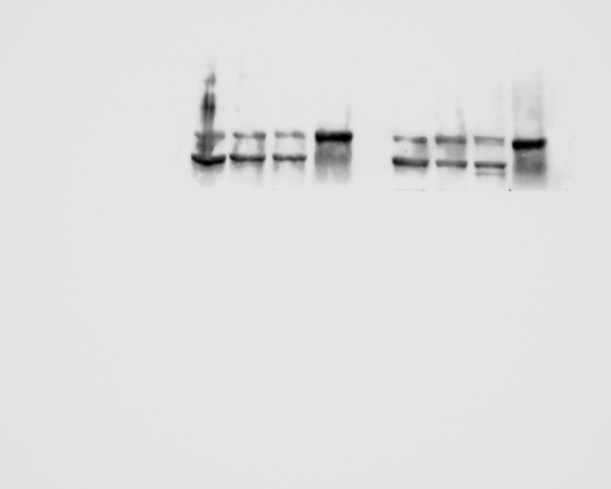


(kDa)

75

50

Cut at 37


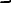

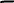


Lanes:

1. Ladder
2. GFP mouse A cortex
3. GFP mouse A cerebellum
4. GFP mouse A spinal cord
5. GFP mouse A liver
6. Ladder
7. GFP mouse B cortex
8. GFP mouse B cerebellum
9. GFP mouse B spinal cord
10. GFP mouse B liver

Notes:

- This membrane was cut at ~37 kDa prior to blocking and antibody hybridization.
- Ladders traced for emphasis.

Anti-GFP

1 2 3 4 5 6 7 8 9 10

(kDa)

Cut at 37

25

20

15


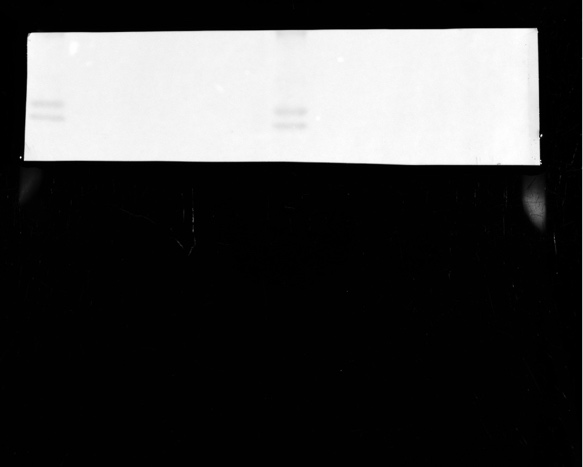

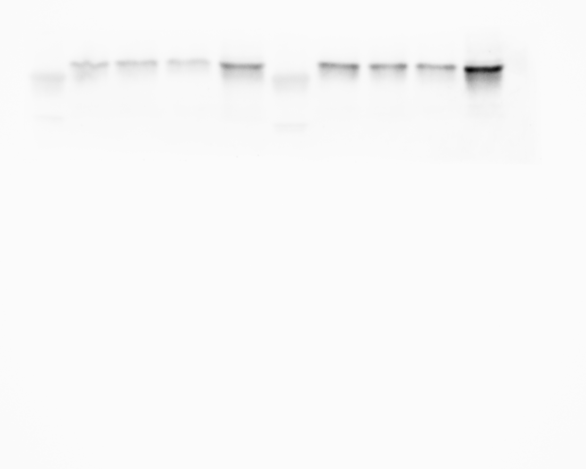


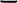

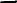


Lanes:

1. Ladder
2. GFP mouse A cortex
3. GFP mouse A cerebellum
4. GFP mouse A spinal cord
5. GFP mouse A liver
6. Ladder
7. GFP mouse B cortex
8. GFP mouse B cerebellum
9. GFP mouse B spinal cord
10. GFP mouse B liver

Notes:

- This membrane was cut at ~37 kDa prior to blocking and antibody hybridization and is the bottom half of Additional Replications- anti- beta-tubulin.
- Ladders traced for emphasis.
